# Monolingual and bilingual infants' attention to talking faces: evidence from eye-tracking and Bayesian modeling

**DOI:** 10.3389/fpsyg.2024.1373191

**Published:** 2024-03-14

**Authors:** Sophie Lemonnier, Benjamin Fayolle, Nuria Sebastian-Galles, Roland Brémond, Julien Diard, Mathilde Fort

**Affiliations:** ^1^Université de Lorraine, PErSEUs, Metz, France; ^2^Laboratoire de Psychologie et NeuroCognition, Univ. Grenoble Alpes, Univ. Savoie Mont Blanc, CNRS UMR 5105, Grenoble, France; ^3^Center for Brain and Cognition, Universitat Pompeu Fabra, Barcelona, Spain; ^4^Université Gustave Eiffel, PICS-L, Marne-la-Vallée, France; ^5^Centre de Recherche en Neurosciences de Lyon, CRNL UMR 5292, Université Lyon 1, Lyon, France

**Keywords:** infant, visual attention, bilingualism, Bayesian modeling, eye-tracking, early language acquisition, talking faces, audiovisual speech perception

## Abstract

**Introduction:**

A substantial amount of research from the last two decades suggests that infants' attention to the eyes and mouth regions of talking faces could be a supporting mechanism by which they acquire their native(s) language(s). Importantly, attentional strategies seem to be sensitive to three types of constraints: the properties of the stimulus, the infants' attentional control skills (which improve with age and brain maturation) and their previous linguistic and non-linguistic knowledge. The goal of the present paper is to present a probabilistic model to simulate infants' visual attention control to talking faces as a function of their language learning environment (monolingual vs. bilingual), attention maturation (i.e., age) and their increasing knowledge concerning the task at stake (detecting and learning to anticipate information displayed in the eyes or the mouth region of the speaker).

**Methods:**

To test the model, we first considered experimental eye-tracking data from monolingual and bilingual infants (aged between 12 and 18 months; in part already published) exploring a face speaking in their native language. In each of these conditions, we compared the proportion of total looking time on each of the two areas of interest (eyes vs. mouth of the speaker).

**Results:**

In line with previous studies, our experimental results show a strong bias for the mouth (over the eyes) region of the speaker, regardless of age. Furthermore, monolingual and bilingual infants appear to have different developmental trajectories, which is consistent with and extends previous results observed in the first year. Comparison of model simulations with experimental data shows that the model successfully captures patterns of visuo-attentional orientation through the three parameters that effectively modulate the simulated visuo-attentional behavior.

**Discussion:**

We interpret parameter values, and find that they adequately reflect evolution of strength and speed of anticipatory learning; we further discuss their descriptive and explanatory power.

## 1 Introduction

During their first year, infants experience the face of their caregivers a lot, everyday (Jayaraman et al., 2015; Jayaraman and Smith, 2019). Such daily experience probably sculpts infant social and language development in a specific manner (Maurer and Werker, 2014; Pascalis et al., 2014). Faces are the source of complex social information, which changes quickly over time and space. For instance, orientations of gaze are essential in conveying identity (Farroni et al., 2007) and emotional cues (Jessen and Grossmann, 2014) as well as in establishing a communicative connection with another individual (Senju and Csibra, 2008). Along with head direction, they also provide directional information for joint attention, which in turn scaffolds language development (Tomasello and Farrar, 1986, see Yurkovic-Harding et al., 2022 for a recent review). The mouth region also provides emotional information, as well as redundant auditory and visuo-articulatory speech cues that can notably be of use in language learning (Munhall and Johnson, 2012). Thus, one may assume that developing an efficient attentional control system that optimizes the processing of talking faces improves how infants acquire their native(s) language(s). The goal of the present paper is to propose a computational model of how the maturation of infants' visual attention control influences infants' gaze orientation to different areas of talking faces.

Growing evidence gathered in the past few decades suggests that developing an efficient attention control system is crucial for language learning (Smith et al., 2014, 2018). Notably, due to the temporal and transient nature of speech signals, the ability to precisely control one's attention to these temporal events appears necessary (de Diego-Balaguer et al., 2016). In the case of talking faces, infants need to learn to flexibly control their attentional resources when interacting with others in order to properly focus on and exploit the task-relevant cues *over time* to boost their language learning. As reviewed below, both sources of information promote early language acquisition.

On the one hand, research has shown that paying attention to the eye region of a face could be a good strategy: for instance, infants are already sensitive to direct gaze (rather than indirect gaze) a few hours after birth (Farroni et al., 2002, 2006), a social signal that establishes the intent to communicate. Infants then develop the ability to control their attention to follow the head and the eye-gaze direction of their social partners during the first year (Senju and Csibra, 2008). This gaze-following ability boosts word-learning performance during the second year, notably by providing crucial referential information (e.g., designating a named object, Brooks and Meltzoff, 2005, 2008, 2015). Importantly, more recent research (Yu et al., 2019) evidenced that infants' sustained attention skills are more predictive of their vocabulary development than infant joint attention skills per se, suggesting that attentional control is a key process in word learning.

On the other hand, paying attention to the mouth of talking faces over time may also be useful. By focusing on the mouth, infants are able to access redundant audiovisual speech cues and potentially better encode the articulatory gestures of their caregiver's face. However, while paying attention to the mouth of talking faces drastically improves how adults recognize words (Fort et al., 2010), infants in their first months do not seem able to take full advantage of this source of multimodal speech signals (Murray et al., 2016). This might be due to the fact that, for young infants, selecting (and sustaining one's attention on) the relevant spatial and temporal information is challenging, as their attentional control system is still underdeveloped. Accordingly, when presented with an audiovisual talking face telling them short stories, 4- and to a lesser extent, 6-month-old infants look more at the eyes than at the mouth region (Lewkowicz and Hansen-Tift, 2012). In this situation, as opposed to the informative talking mouth region, no novel information is provided by the eye region (i.e., constant direct gaze), making it less informative than the mouth over time. As such, we can therefore assume that this early preference for the eyes is probably due to infants' immature attentional control: their attention is automatically drawn to the eye region, due to the high physical saliency of the visual contrast between the white sclera and the darker pupil (Otsuka et al., 2013). As age increases, infants start to shift their attention from the eyes to the more informative talking mouth of the speaker, and sustain it there. Interestingly, a clear mouth preference is observed from around 8 months of age, when their attentional control system becomes clearly functional (de Diego-Balaguer et al., 2016). Further research suggests that from 6 to 8 months, this enriched signal could help them to build their phonetic categories (Teinonen et al., 2008; Ter Schure et al., 2016) and, during the second year, provide support for memorizing word forms in their lexicon (Weatherhead and White, 2017; Weatherhead et al., 2021). Focusing on the mouth of caregivers is also positively correlated with vocal imitation and the onset of word production (Imafuku et al., 2019). These audiovisual speech cues could also contribute to learning one's native language in challenging language environments. For instance, infants growing up in a bilingual environment seem to pay more attention to the mouth region of talking faces than their monolingual peers (Pons et al., 2015; Ayneto and Sebastian-Galles, 2017; Fort et al., 2018; Birulés et al., 2019) (but see also Morin-Lessard et al., 2019; Pejovic et al., 2021 for contrasting results).

In summary, the current research is compatible with the assumption that infants' ability to benefit from the eyes and the mouth region of talking faces could be mediated by attentional control maturation. However, one important parameter to consider is the role of top-down information: as infants gain attentional control to explore and benefit from the information provided by talking faces, they should also increase their knowledge of their native language(s). This newly acquired information should in turn also constrain how infants explore talking faces over time.

To our knowledge, only one study investigated the effect of language background constraints on infant attention to different regions of talking faces over time, as a function of accumulated information (Fort et al., 2018). In this study, the authors tested how monolingual and bilingual infants, at the beginning of their second year, update their pattern of selective attention to talking faces as a function of the information provided in the eyes or the mouth region at each trial. At each trial, infants saw a speaker producing a short sentence. After each sentence, the speaker would produce a speech-irrelevant movement [an EyeBrow raise (EB) or a Lip Protrusion (LP) movement]. At 15 months, all infants preferred to look overall more at the mouth than at the eye region of the speaker while she was talking (during the sentences). This first result may be due to the fact that all infants of this age had an attentional control system mature enough to overcome their automatic orientation to the salient eye region. However, only monolingual infants in the “eyebrow-raised” condition learned, from trial to trial, to decrease their pattern of attention to the mouth during the sentence, to anticipate the eyebrow-raise movement. At 18 months, bilingual infants, as well as their 15 month-old monolingual peers, disengaged from the talking mouth to learn to anticipate the eyebrow-raise movement at the end of each sentence. This study shows that the exploration of talking faces results from a subtle combination of language environment constraints (such as bilingualism), attentional control maturation, and the salience/relevance of the information provided by the eyes and the mouth over time. However, this study was not able to clearly tease apart the respective roles of attentional maturation and bilingual experience. The goal of the present research is to go one step further in that direction by providing a modeling framework of how the maturation of visual attention could predict infant visual exploration of talking faces.

In order to propose the architecture of a computational model of the orienting of visual attention control, we relied on the current literature on visual attention in adults and infants. Visual attention has been classically defined as being guided by a combination of two components: a bottom-up and a top-down system (Carrasco, 2011; Petersen and Posner, 2012). The bottom-up component depends on environmental, exogenous factors, such as object saliency (Koch and Ullman, 1985; Hughes and Cole, 1986). It allows the automatic detection and orientation of visual attention toward physically salient stimuli in the environment. It is sustained by sub-cortical structures, and becomes mature by the age of 6 months (Colombo, 2001).

The top-down component depends on endogenous factors, such as the task, goals and knowledge of the individual (Yarbus, 1967; Einhäuser et al., 2008). It supports the volitional control of spatial and temporal visual attention as a function of task constraints and the observer's motivations. It involves attending to relevant information while at the same time inhibiting irrelevant or distracting information (Ansorge and Fuchs, 2012). This ability is crucial for maintaining one's attention over time and matures during infanthood and childhood (Ansorge and Fuchs, 2012; Downes et al., 2018). As opposed to the bottom-up system, the top-down component is linked to the development of frontal and pre-frontal cortical structures, which develop later than other brain areas; it only starts to be functional in the second half of the first year (Richards, 2003; de Diego-Balaguer et al., 2016; Oakes and Amso, 2018).

The recent consensus in the literature about adult visual attention is that a third “selection history” component is to be distinguished from the bottom-up and top-down components (Theeuwes and Failing, 2020). This selection history provides mechanisms and representations such that attention orientation depends on previous visual selections (Awh et al., 2012). It depends on the observer's previous knowledge of the scene and is independent of its bottom-up visual features. This component encompasses both inhibition of return and more general, knowledge-related mechanisms (e.g., not orienting toward an already well-known region). In previous models, it was sometimes previously considered as part of the top-down component. However, it needs to be distinguished, first, from the top-down component since it is fast, automatic and effortless, contrary to other goal-guided pieces of knowledge and representations related to volitional attention, and second, from the bottom-up component as well, as it is based on reward (Anderson et al., 2011) and priming (Theeuwes and Van der Burg, 2013).

In contrast to experimental data and theoretical accounts advocating for a three-component model of visual attention, most computational models in adult visual attention literature only considered the bottom-up component. This is probably due to the fact that these models are usually based on very specific eye-tracking data from visual search tasks (Borji and Itti, 2012; Kotseruba and Tsotsos, 2021). However, several studies have shown the importance of considering the top-down and selection history components in their architecture to satisfactorily predict visual attention in ecological situations (Tatler et al., 2005; Henderson et al., 2009; Wolfe et al., 2010; Theeuwes and Failing, 2020). Indeed, when the environment is dynamic and the task is similar to free-viewing, bottom-up models alone often cannot explain observations, whereas multi-component models of visual attention are more successful (e.g., Borji et al., 2012). Moreover, to the best of our knowledge, there are no experimental data in the infant literature involving these three components of visual attention.

The goal of the present research is to provide a computational, three-component model of visual attention. We developed a probabilistic model that simulates the infants' visual attention control, and its maturation when exploring and learning from talking faces. More precisely, we introduce the PROTOBOT model (PRObabilistic model of visual attention with TOp-down, BOttom-up and Temporal history), to predict which area of the face (eyes, mouth) is more susceptible to be attended to, over time. It enables us to weight the contribution of its three components: the bottom-up component, which is driven by the physical salience of different parts of the face over time, the top-down component, which is driven by the task demands, and the selection history component, which constrains the infants' next visual explorations by taking into account previous explorations.

The model provides predictions of infant visual exploration of talking faces as a function of the varying physical saliency of the stimulus and of the presence or absence of a dual-language environment. Last but not least, it also simulates the maturation of infants' top-down attentional control by varying the weights of bottom-up and top-down components, accounting for different behavior in the allocation of attention in different age groups. Model parameters were adjusted, then model predictions were compared with previously collected eye-tracking data from monolingual and bilingual infants of 12, 15 and 18 months of age when exploring talking faces providing time-varying information in the eyes or the mouth region that changes over time. More precisely, since part of the data (15 and 18 months) has been published previously (Fort et al., 2018), the original contributions of the present paper are the PROTOBOT model, the 12-month-old infant data, and the comparison of model predictions to behavioral data (12, 15 and 18 months).

## 2 Materials and methods

### 2.1 Participants

An overall total of 269 healthy full-term infants participated in this study. The data from 89 infants were excluded from the final analysis (see details per age and language group below), so that the N total included in the final analyzed sample = 180 infants (i.e., 80 12-month-old, 80 15-month-old, and 20 18-month-old). They were all recruited from private clinics in Barcelona. Thus, it can be considered that they were all raised in mid or mid-upper socio-economic status families. All of them did the same eye-tracking task presented below. All parents completed a language questionnaire adapted from Bosch and Sebastián-Gallés (2001) to assess their infant's language background. All the experiments presented in this manuscript followed the General Data Protection Regulation (GDPR). This means that methods were performed in accordance with the relevant guidelines and regulations for testing human participants in the European Union. Notably, informed written consent for their child to participate in the present study was required from parents or legal representatives.

The infants were split into two experimental conditions (presented below): the Eyebrow-raise condition and the Lip-protrusion condition. The distribution of infants by experimental design is summarized in Table 1. The behavioral results from the 15- and 18-month-old have already been published (Fort et al., 2018). In what follows, therefore, the characteristics of 12-month-old infants are discussed in more detail.

**Table 1:** Distribution of infants by age group; whether they were considered monolingual or bilingual; their numbers (N); their numbers by gender (N girls); their distribution by the two experimental conditions (Eyebrow-raise and Lip-protrusion); and whether the data have already been published.

**Age**	**Bilingualism**	**N**	**N girls**	**Condition**	**Previously published result**
12 m	Monolingual	40	21	20 / 20	
	Bilingual	40	23	20 / 20	
15 m	Monolingual	40	20	20 / 20	(Fort et al., 2018), Experiment 1
	Bilingual	40	31	20 / 20	(Fort et al., 2018), Experiment 1
18 m	Bilingual	20	10	20 / 0	(Fort et al., 2018), Experiment 2

Forty of the 12-month-old infants from the final sample were raised in a Spanish-Catalan bilingual environment (bilinguals), and were exposed to their non-dominant second language at least 20% of the time (age range: 345–388 days; mean age: 368 days; 23 girls). Half of the bilingual infants were randomly assigned to the Eyebrow-raise condition (*N* = 20, 11 girls, *N* = 15 Catalan dominant, exposure to the dominant language = 67%, SD = 10%), while the other half participated in the Lip-protrusion condition (*N* = 20, 12 girls, *N* = 11 Catalan dominant, exposure to the dominant language = 65%, SD = 8%). Crucially, the infants in the Eyebrow-raise condition neither differed in terms of age nor of language exposure from the infants in the Lip-protrusion condition (all *p* > 0.05). The data from 22 additional infants were excluded from the final analyzed sample due to being exposed to a third language (*N* = 1) or due to total time looking at the screen being less than 50% (*N* = 10), insufficient number of trials (*N* = 4) or failure to calibrate (*N* = 7).

The other forty 12-month-old infants from the final sample were considered as monolinguals as they were exposed to Catalan or Spanish at least 85% of the time (age range: 351–396 days; mean: 372 days; 19 boys). Twenty of them participated in the Eyebrow-raise condition (11 girls, 15 Catalans, mean language exposure to the dominant language = 94%, SD = 5%), while the other 20 were tested in the Lip-protrusion condition (10 girls, 18 Catalans, mean language exposure to the dominant language = 94%, SD = 6%). The monolinguals in the Eyebrow-raise condition did not differ from the ones in the Lip-Protrusion condition in terms of language exposure and age (in days, all *p* > 0.05). The data from 24 additional infants were excluded from the final analysis due to the total time looking at the screen being less than 50% (*N* = 18), insufficient number of trials (*N* = 2), and failure to calibrate the eye-tracker (*N* = 4). Importantly, the monolinguals in both conditions were comparable within their own group and to both groups of bilinguals in terms of age (in days, all *p* > 0.05).

Forty of the 15-month-old were Spanish-Catalan bilinguals, and the other forty were Spanish or Catalan monolinguals (see Fort et al., 2018, for more details). Half of the 15-month-old bilinguals and monolinguals participated in the Eyebrow-raise condition, while the other half participated in the Lip-protrusion condition. Fifteen-month-old were equivalent in terms of age across conditions and language groups (all *p* > 0.05). The group of 20 18-month-old were all Spanish-Catalan bilinguals. For this age group we only focused on the critical Eyebrow-raise condition. The reason why they were only tested in the Eyebrow-raise condition is detailed in Fort et al. (2018). Within bilingual and monolingual groups, language exposure was equivalent across conditions and age groups (all *p* > 0.05). Overall, we used the same sample size (namely 20 infants per language group, age and condition) than previous seminal studies (Lewkowicz and Hansen-Tift, 2012; Pons et al., 2015).

### 2.2 Stimuli and recordings

The stimuli and recordings were exactly the same as in Fort et al. (2018) and are all available on OSF (https://osf.io/mwfhc/files). The Speech Events consisted of video recordings of 19 different sentences (e.g., “Cada día canto” *Everyday I sing*, fixed length: 6 syllables, duration range: 1,180–2,200 ms, mean duration: 1,800 ms). The Non-speech Events were video recordings of the speaker raising her eyebrows (Eyebrow-raise condition) or protruding her lips (Lip-protrusion condition). The durations of both Non-speech Events were identical (fixed duration: 1,880 ms). The complete set of stimuli was produced by a bilingual Spanish-Catalan Caucasian female speaker. She maintained her head in the same position for the duration of the recordings. The Speech Events were recited using adult-directed-speech and recorded both in Spanish and in Catalan (19 sentences in each language), separately from the Non-speech Events. All video-clips were then encoded in an mpeg video format with Adobe Premiere CS3. The Speech Events were then combined with the Non-speech Events in the same video file using Adobe Premiere. The transition between the Speech Events and the Non-speech Events was almost imperceptible.

### 2.3 Procedure

The procedure was the same as in Fort et al. (2018). Infants were seated on their parents' lap in a quiet room 60 cm away from a 1,080×1,920 screen. The stimuli were played with custom-made software using MATLAB (version 7.11; Mathworks, Natick, MA, USA), the MATLAB Psychtoolbox and Tobii Analytics Software Development Kit (Tobii Analytics SDK; Tobii Technology AB, Danderyd, Sweden). The auditory component of the stimuli was displayed at a comfortable hearing level (at 65 dB) with a sampling frequency of 44,100 Hz. The video component was displayed at 25 frames/s. Infants' eye movements were recorded by a Tobii TX300 stand-alone eye tracker (Tobii Technology AB, Danderyd, Sweden, sampling rate: 300 Hz).

Before the experiment, an infant-friendly 5-point calibration procedure (one central point and one at each corner of the screen) was performed. The calibration was reiterated until at least three valid points were obtained. The experiment consisted of one dummy trial followed by 19 test trials. In each trial, the speaker's face (zoomed in to cover the top of her head to the base of her neck) appeared in a naturalistic size in terms of the infants' distance from the screen, in front of a neutral background. The dummy trial consisted of a brief video of the speaker introducing herself smiling at the infant and producing a short warming up sentence “Hola, ¿Qué tal?” *Hi, how are you?* in Spanish or in Catalan (same sentence in both languages). The next 19 test trials all started with an audiovisual attention-getter (looming object available in the Tobii software) to attract the infants' attention to the center of the screen. As soon as infants looked at this attention-getter for 1 s, a video of the speaker producing a Speech Event followed by a Non-speech Event started. For half of the participants, the Non-speech Event was the speaker systematically raising her eyebrows without moving her lips (Eyebrow-raise condition). For the other half, she systematically protruded her lips without moving her eyebrows (Lip-protrusion condition).

Infants were randomly assigned to the Eyebrow-raise or to the Lip-protrusion condition. The videos were played in the infant's native language or most dominant language in their environment. The order of Speech Events was pseudo-randomized, across 10 lists, each list counterbalanced between participants. The whole experiment (calibration phase then 1 dummy trial followed by 19 test trials) lasted about 5 min.

## 3 Data analysis

### 3.1 Behavioral data

**Figure 1:**
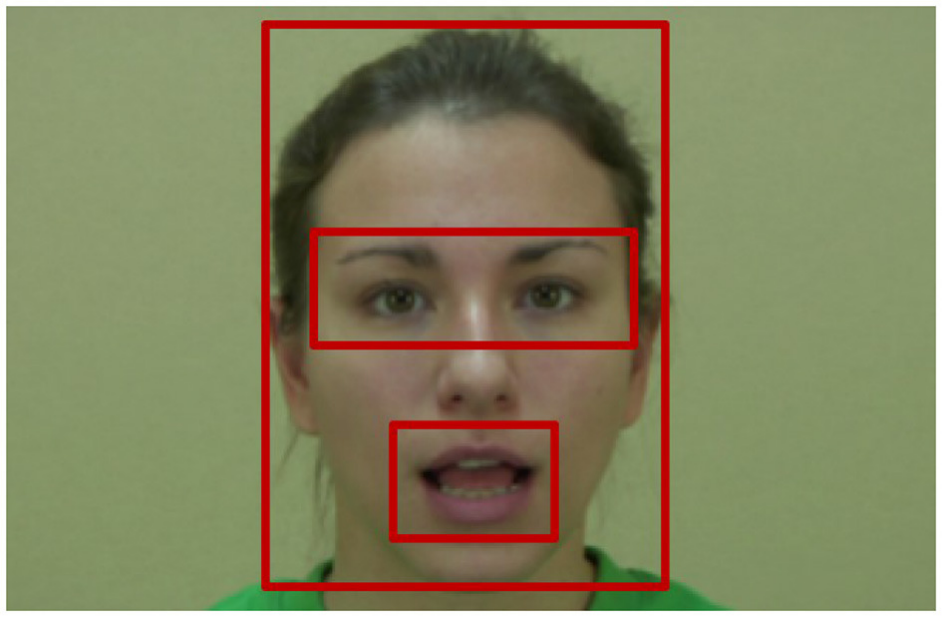
Illustration of the different Areas Of Interest (AOIs).

We used the exact same pre-processing and facial landmarks as in Fort et al. (2018). Two rectangular Areas Of Interest (AOIs) were defined: one around the eyes and eyebrows and one around the mouth of the speaker (see Figure 1). A custom-made program (Matlab 7.11, Tobii Analytics SDK) was used to determine for each trial and each participant, whether each data point collected by the eye-tracker fell into one of these two AOIs, in the rest of the face (third AOI) or anywhere else including away from the screen (fourth AOI). Because of small duration differences between stimuli, we noted time in normalized frames, with Speech Events and Non-speech Events normalized separately, using the resample function in Matlab. In other words, time instant 50% of the Speech Event refers to the mid-point of the speech sequence, time instant 0% of the Non-speech Event to its beginning, etc. The proportion of total looking time (PTLT) to each AOI was then computed for each participant by dividing the total looking time (TLT) at each AOI by the total looking time at the whole face. Note that the TLT for the fourth AOI (anywhere else) is always equal to 0 during the data analysis, which gives the following equation:


(1) $$\begin{array}{lll} PTLT_{\text{Eyes}} & = & \frac{TLT_{\text{Eyes}}}{TLT_{\text{Eyes}}+TLT_{\text{Mouth}}+TLT_{\text{Rest of the Face}}}. \end{array}$$

We performed separate analyses on two different windows of analysis: detection window and anticipatory window (see Figure 1 in Fort et al., 2018). The detection window corresponded to the whole duration of the Non-speech Event. It was designed to ascertain whether infants could detect each type of Non-speech Event during its presentation. The anticipatory window corresponded to the last 50% of the Speech Event. It was designed to ascertain whether infants learned to anticipate the appearance of the Non-speech Event during the last 50% of the Speech Event. For each of these windows, we computed the mean Eyes- and Mouth-PTLT scores. We then computed for each participant, their mean Eyes-PTLTs *minus* their mean Mouth-PTLTs to obtain an individual preference score. The data were not filtered, transformed or trimmer further than what was described in this section or in the participant inclusion criteria.

### 3.2 Bayesian model

In this section, we describe PROTOBOT, the Bayesian model that we developed to simulate eye movement trajectories during observation of a talking face. We present the model in three steps: first, we provide an overall description of the model architecture and its main features, second, we detail each of its components and outline some of their mathematical definitions, and third and finally, we show how applying Bayesian inference in the model yields probabilistic expressions for simulating eye movement trajectories.

#### 3.2.1 Overall architecture of the model

**Figure 2:**
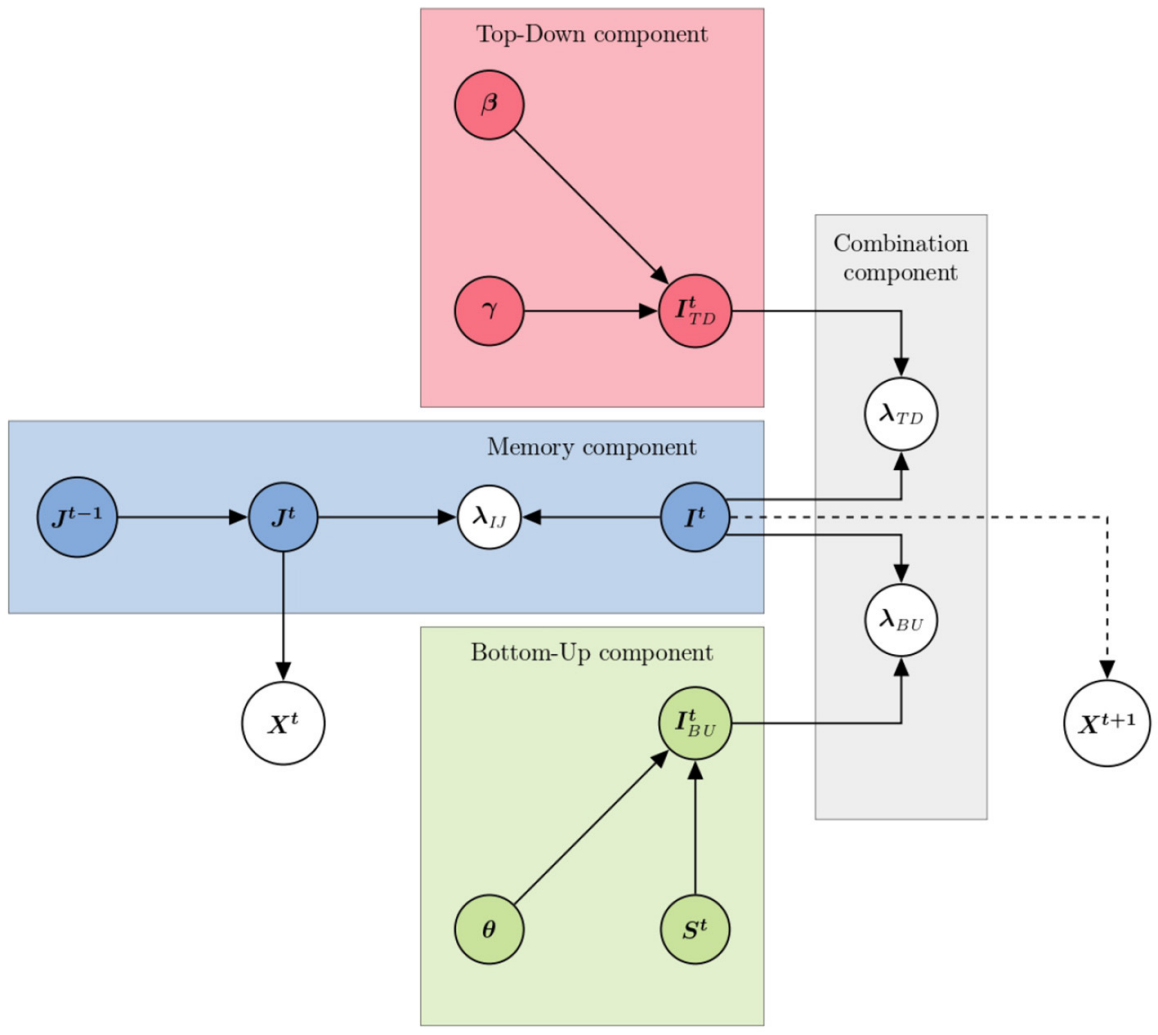
Graphical representation of the model's architecture. The graph follows the usual convention for probabilistic graphical models: each node represents a variable, and arrows illustrate probabilistic dependencies. The dashed arrow indicates random sampling. Colored rectangles delineate the components of the model.

The two main sources of information involved in controlling visuo-attentional positions and displacements are, as argued above, sensory input and task-related knowledge. In our model therefore (see Figure 2), two components relate to these. The first concerns the extraction, from the sensory input and in a bottom-up manner, of information about which position, in the visual scene, would be useful to visit. The second, in a top-down manner, provides information about which position, according to regularities and known constraints of the task or tasks, to visit. We of course refer to these components as the “bottom-up” and “top-down” components, respectively.

The third source of information we consider is the history of past visuo-attentional positions, so that the model avoids visiting a given position of the visual scene for too long, or going back too early to a previously visited position. In the model, each component provides a probability distribution over positions to visit, assigning high probability values to desirable positions; in contrast, the memory component, by tracking previously visited positions, represents positions to avoid. Therefore, the memory component also features an “inversion mechanism,” to translate the probability distribution of desirable positions into the probability distributions of positions to avoid.

These three main components of the model are ultimately concerned with providing probability distributions over positions in the visual scene, to “attract” future visuo-attentional displacements. The visual scene is decomposed into 4 areas of interest (AOIs), respectively covering the eyes of the talking face, the mouth of the talking face, the rest of the face (i.e., portions of the face outside of the eye and mouth AOIs; noted *RoF* for “rest of face” in the following) and the rest of the image (i.e., portions of the image outside of the three other AOIs; noted *Other* in the following). Several probability distributions of the model, therefore, will be defined over “zones,” that is to say, a 4-case discrete space with zones equating the AOIs defined in Figure 1, and such probability distributions represent “how attractive each AOI is to the eye.” This is irrespective of the features that are detected to explain this attraction, or of task constraints that explain why visiting this or that AOI would be useful; in other words, the model is “representationally agnostic” with respect to the contents of visual processing, and it merely models how different components of visual processing interact to vote for or against future visuo-attentional displacements.

The fourth and final component of the model is a “combination component.” to combine probability distributions over positions to visit provided by the bottom-up, top-down, and memory components. The overall architecture of the model is illustrated in Figure 2. Variables (which are accompanied by probability distributions in the model) and parameters (which have values explored in a grid search manner in our experiment) of the model are listed in Table 2. We now introduce the mathematical definitions of each component, in turn.

**Table 2:** Table of variable and parameters of the model (see Equation 6).

**Symbol**	**Type**	**Domain**	**Interpretation**	**Values in grid-search exploration**
**Top-Down component**				
**β**	**Parameter**	**ℝ^+^**	**Strength of TD learning**	**[0.1, 0.5, 1, 2, 3, 4, 5]**
γ	Parameter	ℝ^+^	Speed of anticipation	[0.1, 0.5, 1, 1.5, 2]
$I_{TD}^{t}$	Variable	Zones	Attraction to zones according to TD	
**Memory component**				
*J*^*t*−1^, *J*^*t*^	Variable	Zones	Zones recently visited	
*X* ^*t*^	Variable	Img coord	Eye position	
*I* ^*t*^	Variable	Zones	Attraction to zones according to	
			The memory component	
λ_*IJ*_	Variable	Boolean	Coherence variable for knowledge inversion	
**Bottom-Up component**				
$I_{BU}^{t}$	Variable	Zones	Attraction to zones according to BU	
*S* ^*t*^	Variable	Image	Saliency maps	
θ	Parameter	[0, 1]	Weight of the static saliency map	[0, 0.05, 0.1, 0.25, 0.5, 0.75, 1]
**Combination of components**				
λ_*BU*_, λ_*TD*_	Variable	Boolean	Coherence variables for component combination	

Columns provide variable and parameter symbols, interpretations and domains. Additionally, for parameters, types, values for the grid search exploration of simulations are provided. The “zones” domain refers to the 4-case discrete space {Eyes, Mouth, RoF, Other}, referring to the 4 AOIs.

#### 3.2.2 Bottom-up component

**Figure 3:**
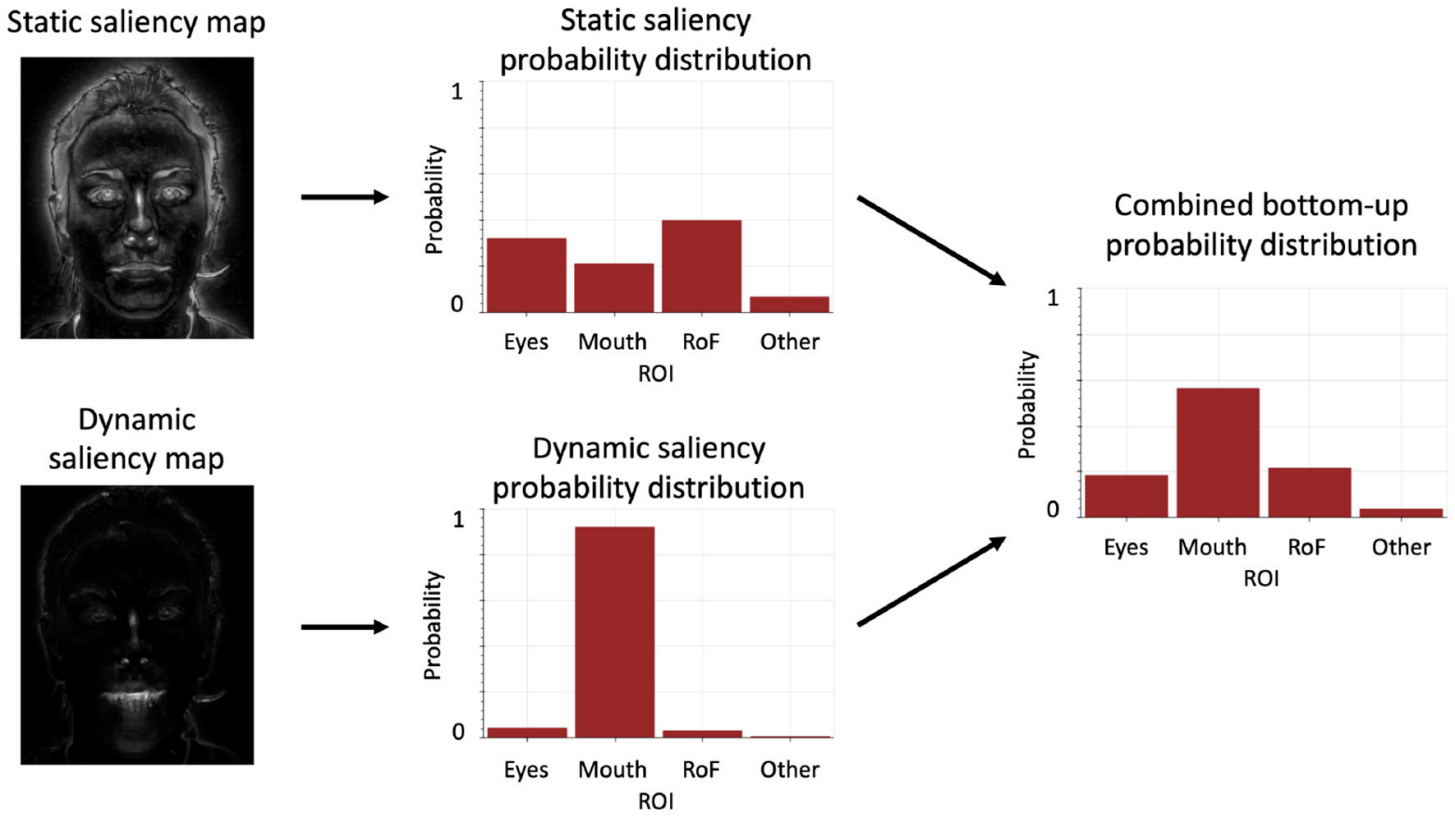
Schematic computation flow of the bottom-up component.

The first component we describe, and the one that should be the most familiar, concerns the extraction of salient visual features from the stimulus. It relies on classical, off-the-shelf methods, and involves computing two saliency maps, based respectively on static and dynamic features. It is illustrated in Figure 3.

First, we consider the visual stimulus as a sequence of gray-scaled images. The static saliency map is computed by an algorithm that implements ^1^ the classical method by Itti et al. (1998). This method involves computing, for each pixel of an input image, the intensity difference between the pixel and its neighborhood, in a “center-surround” fashion inspired by neuronal visual processing. This provides high saliency to contours of objects in the image, and low saliency to inside regions (see Figure 3 top left). The dynamic saliency map is also computed in a classical manner (Frank et al., 2009). We first compute the squared intensity of each pixel in each frame of the input video, and then the pixel-based difference between successive frames. This provides high saliency to moving portions, and low saliency to static portions of the video (see Figure 3 bottom left).

We then aggregate the saliency maps in each AOI, by averaging each measure in the predefined image area. This provides two probability distributions, representing which AOI is most likely to contain salient static or dynamic features. These are combined by a weighted average to provide the overall probability distribution about salient visual features. In our probabilistic model, this final distribution provided by the bottom-up component can thus be noted as $P\left(I_{\text{BU}}^{t}|S^{t}\;\theta \right)$, with *S*^*t*^ the saliency maps at time *t* and θ the weighting parameter of the static saliency map in the combination. The free parameter θ (varying between 0 and 1 in the following experiments), can be interpreted as follows: a value of θ close to 1 translates into an increased preference for the static saliency components (e.g., with our stimuli of a talking face, a preference for static saliency, i.e., the eye region), and a value close to 0 into an increased preference for dynamic saliency components (e.g., with our stimuli, a preference for movement, i.e., the eyes or mouth region).

#### 3.2.3 Memory component

The second component we describe, the memory component, is in charge of both maintaining and updating a selection history dynamically during simulation, that is to say, keeping track of how often zones were recently visited, and an inversion mechanism, so that zones recently visited become less attractive. Mathematically, this is implemented with two probability distributions over zones: one over variable *J*^*t*^, for the selection history, and the other one over variable *I*^*t*^ for the result after inversion. More precisely, a probability distribution over variable *J*^*t*^ represents zones recently visually processed, and updates it as a function of the latest eye position *X*^*t*^. Zones with high probability in this distribution over *J*^*t*^ indicate recently visited zones. The inversion mechanism translates this distribution over *J*^*t*^ into a distribution over *I*^*t*^, to indicate zones to go and visually explore. We describe these two mechanisms successively.

The selection history involves variables *J*^*t*−1^ and *J*^*t*^, to encode the evolution of the probability distribution over visited zones over time. Updating this distribution involves two steps. First, the previous distribution *P*(*J*^*t*−1^) is “slightly diluted,” to account for both memory decay and information obsolescence, which occurs in the context of visually observing dynamic scenes. Mathematically, this dilution involves the probability distribution *P*(*J*^*t*^∣*J*^*t*−1^); in the terminology of probabilistic models such as Hidden Markov Models (HMM) or Bayesian Filters, this is a dynamic or temporal model of information evolution. It is defined by a conditional probability distribution, with a high, almost 1 probability that information is maintained (*P*([*J*^*t*^ = *j*^*t*^]∣[*J*^*t*−1^ = *j*^*t*−1^]) ≈ 1 when *j*^*t*^ = *j*^*t*−1^), and a uniformly distributed probability that information decays (*P*([*J*^*t*^ = *j*^*t*^]∣[*J*^*t*−1^ = *j*^*t*−1^]) = ϵ when *j*^*t*^ ≠ *j*^*t*−1^, with ϵ a small, empirically set value). In the absence of external stimulation, this would yield a gradual decay of probability distribution over zones *P*(*J*^*t*^) back into its initial, resting state (in our case, a uniform probability distribution).

The second step to update the selection history is to take into account the current eye position *X*^*t*^. Mathematically, again using classical terminology and the usual structure of temporal probabilistic models, this is defined in the observation model *P*(*X*^*t*^∣*J*^*t*^). The observation model assigns a higher probability to the zone viewed at time *t*, corresponding to the eye position *X*^*t*^, to reflect the fact that information has just been retrieved from this zone. In the ensuing calculations, this is reflected in the selection history by an increase in the probability in the zones viewed, representing the fact that these zones have recently received visual processing resources. We note that the memory component is agnostic to the actual “contents” of visual processing; indeed, our model, overall, is concerned with modeling *where* visual processing and the eye go, irrespective of *what* is visually processed.

The computed selection history thus represents which zones were recently visited, and thus should be avoided. The second mechanism of the memory component “inverts” this probability distribution, so that recently visited zones are assigned low probability. To achieve this, we transform the probability distribution over *J*^*t*^ into a probability distribution over variable *I*^*t*^, using a specific, custom mathematical construct, called a coherence variable. The one featured in the memory component is noted λ_*IJ*_.

A coherence variable (Gilet et al., 2011; Bessière et al., 2013) is a probabilistic binary variable, attached to a specific probability distribution. Overall, coherence variables can be seen as “versatile connectors,” to link together portions of probabilistic models in various ways. For instance, they can be used to control information flow in an all-or-none fashion in a model (Gilet et al., 2011), to study portions of the model in isolation (Laurent et al., 2017), or to temporally control which portion of a model receives sensory stimulation (Nabé et al., 2021; Nabé et al., 2022). They can also be “controlled,” yielding gradual control of information flow in the model (Ginestet et al., 2019). Observing the states of coherence variables instead of controlling them yields pattern recognition mechanisms, based on similarity measures between predicted and observed probability distributions (Steinhilber et al., 2022).

It can be shown that, when coherence variable λ_*IJ*_ is set to 0, computing the probability distribution over *I*^*t*^ yields:


(2) $$\begin{array}{lll} P\left(\left[I^{t}=i^{t}\right]|\left[\lambda_{\text{IJ}}=0\right]\right) & = & \frac{1-P\left(\left[J^{t}=i^{t}\right]\right)}{z-1}\;. \end{array}$$

The demonstration is provided in Appendix S1.

This is interpreted as the desired inversion operator: in this computation, the probability of zone *i*^*t*^ according to $P\left(\left[I^{t}=i^{t}\right]|\left[\lambda_{\text{IJ}}=0\right]\right)$ is high when it is low according to *P*([*J*^*t*^ = *i*^*t*^]), and vice-versa, thanks to the [1−·] operator. The denominator merely implements re-normalization, to ensure that the constraints of probabilistic inference are satisfied.

#### 3.2.4 Top-down component

The third main component of the model is the top-down component, concerned with representing knowledge about the task at hand, and where visual processing should be preferably allocated. Mathematically, this involves a probability distribution over variable $I_{\text{TD}}^{t}$.

The first ingredient in this probability distribution is a preference for the Eyes and Mouth zones, and a small probability for the RoF and Other zones:


(3) $$\begin{array}{lll} P\left(\left[I_{\text{TD}}^{t}=\text{Eyes}\right]\right) & = & p_{\text{Eyes}}^{t}=.45\\ P\left(\left[I_{\text{TD}}^{t}=\text{Mouth}\right]\right) & = & p_{\text{Mouth}}^{t}=.45\\ P\left(\left[I_{\text{TD}}^{t}=\text{RoF}\right]\right) & = & p_{\text{RoF}}^{t}=.05\\ P\left(\left[I_{\text{TD}}^{t}=\text{Other}\right]\right) & = & p_{\text{Other}}^{t}=.05\;. \end{array}$$

The second ingredient in the top-down component is a learning mechanism, to model the ability to acquire knowledge about the temporal patterns of remarkable events in the visual scene. More precisely, in the context of the visual exploration of talking faces by infants in the experiment, we consider the Eyebrow-Raise condition. In this condition, the visual scene is first a Speech Event systematically followed by a Non-speech Event in which the speaker raises her eyebrows. We recall that, at the first trial, this movement should be considered unexpected, and thus possible eye movements toward the eyes should only happen after eyebrow movement happens in the stimulus video. We do not model the learning mechanism explicitly, and instead, we model the result of learning; to do so, we provide the model with the means to anticipate this Non-speech Event, so as to favor observation of the eye region when it happens, and slightly before it happens in the case of successful anticipation.

Mathematically, this is performed by modulating the probability value $p_{\text{Eyes}}^{t}$ as a function of trial number *T* and time instant *t*. First, we increase $p_{\text{Eyes}}^{t}$ as trials *T* increase, to represent a higher propensity to observe the eyebrow-raise movements as they are repeated. We introduce a free parameter β (varying between 0, representing an absence of learning, and 5, representing strong learning, in the following experiments), to define


(4) $$\begin{array}{l} p_{\text{Eyes, Learn}}^{t}=\left(\beta T+1\right)p_{\text{Eyes}}^{t}\;. \end{array}$$

Given this increased probability value, the probability distribution over zones in the top-down component $P\left(I_{\text{TD}}^{t}\right)$ is obtained by re-normalization. The resulting model is easily interpreted: the higher the value of β, the more it is likely the eye region is observed by the model.

Second, and to model possible anticipation in a straightforward manner, we define the time instant *t*_*a*_ at which the probability to observe the Eyes zone switches from $p_{\text{Eyes}}^{t}$ to $p_{\text{Eyes, Learn}}^{t}$ also as a function of trial number *T*, and of a second free parameter γ (varying between 0 and 2 in the following experiments):


(5) $$\begin{array}{l} t_{a}=t_{\text{ER}}-\gamma T\;, \end{array}$$

with *t*_*ER*_ the time instant of the beginning of the eyebrow-raise movements. In other words, when γ = 0, there is no anticipation, and the increased preference for the eye region only occurs after the eyebrow-raise movement is visible in the stimulus. For large values of γ on the other hand, the increased preference for the eye region precedes the eyebrow-raise movement.

#### 3.2.5 Combination component

The fourth and final component implements a mechanism to combine the probability distributions over zones respectively provided by the top-down, memory and bottom-up components. Mathematically, it features two coherence variables, λ_*TD*_ and λ_*BU*_, and the associated probability distributions $P\left(\lambda_{\text{TD}}|I_{\text{TD}}^{t}I^{t}\right)$ and $P\left(\lambda_{\text{BU}}|I_{\text{BU}}^{t}I^{t}\right)$, connecting variables $I_{\text{TD}}^{t}$ and *I*^*t*^ on the one hand, and $I_{\text{BU}}^{t}$ and *I*^*t*^ on the other hand.

The ensuing computations assume that these two coherence variables are set to 1, which, contrary to their previous use in the memory component as an “inversion” mechanism, implements their usual behavior as closed “Bayesian switches” (Gilet et al., 2011). In other words, they connect the three components together, which mathematically combines the probability distributions they provided by multiplying them.

#### 3.2.6 Mathematical definition of the full model

The model is defined by its joint probability distribution, that is to say, the probability distribution over the conjunction of all variables it features. It is defined by:


(6) $$\begin{array}{l} P\left(I_{\text{TD}}^{t}\beta \gamma \lambda TDJ^{t-1}J^{t}\lambda IJI^{t}\lambda BUI_{\text{BU}}^{t}S^{t}X^{t}\theta \right)\\ =P\left(I_{\text{TD}}^{t}|\beta \gamma \right)P\left(\beta \right)P\left(\gamma \right) \end{array}$$

(7) $$\begin{array}{l} P\left(J^{t-1}\right)P\left(J^{t}|J^{t-1}\right)P\left(X^{t}|J^{t}\right)P\left(I^{t}\right)P\left(\lambda IJ|I^{t}J^{t}\right)\\ P\left(I_{\text{BU}}^{t}|S^{t}\theta \right)P\left(S^{t}\right)P\left(\theta \right)\\ P\left(\lambda TD|I_{\text{TD}}^{t}I^{t}\right)P\left(\lambda BU|I_{\text{BU}}^{t}I^{t}\right). \end{array}$$

We have organized terms in the decomposition to correspond to the structure illustrated graphically in Figure 2: the first line corresponds to terms of the top-down component, the second to terms of the memory component, the third to terms of the bottom-up component, and the fourth and final line to terms of the combination component.

#### 3.2.7 Probabilistic inference in the model

The model being fully defined, we now turn to using it to simulate eye movement trajectories. To do so, we compute, at each time step *t*, the probability distributions over zones, given the sensory input and the recurrence term, that is to say, the probability distribution over zones at the previous time step *t* − 1. To be more precise, we apply Bayesian inference in the model, and compute:


(8) $$\begin{array}{l} P\left(I^{t}|\left[\lambda_{\text{BU}}=1\right]\;\left[\lambda_{\text{TD}}=1\right]\;\left[\lambda_{\text{IJ}}=0\right]X^{t}S^{t}\;\beta \;\gamma \;\theta \right)\\ \propto P\left(I_{\text{TD}}^{t}|\beta \;\gamma \right)P\left(I_{\text{BU}}^{t}|S^{t}\;\beta \right)P\left(I^{t}|\left[\lambda_{\text{IJ}}=0\right]X^{t}\right) \end{array}$$

(9) $$\begin{array}{l} \propto P\left(I_{\text{TD}}^{t}|\beta \;\gamma \right)P\left(I_{\text{BU}}^{t}|S^{t}\;\beta \right)\left[1-\frac{P\left(X^{t}|J^{t}\right)}{P\left(X^{t}\right)}\underset{J^{t-1}}{\sum }P\left(J^{t}|J^{t-1}\right)P\left(J^{t-1}\right)\right]\;. \end{array}$$

The complete derivation is provided in Appendix S2.

This computation can be interpreted as follows. First, we compute and update the probability distributions over zones with variables *J*^*t*−1^ and *J*^*t*^. This is done with the last factor in Equation 9. The innermost summation is the classical application of the temporal model over variable *J*^*t*^, which, in our case, only performs a small dilution of the previous probability distribution over *J*^*t*−1^. The result of the summation is then multiplied by the observation model *P*(*X*^*t*^∣*J*^*t*^), modeling acquisition of sensory information as a function of eye position. The resulting distribution is then “inverted,” mathematically by the [1−·] operation and ensuing renormalization. Therefore, the third factor in Equation 9 can also be written as $P\left(I^{t}|\left[\lambda_{\text{IJ}}=0\right]X^{t}\right)$, that is, the probability over zones based only on the current state of the memory component.

The rest of Equation 9 describes how this probability distribution is combined with probability distributions from the bottom-up and top-down components. Bayesian inference and the properties of coherence variables λ_*BU*_ and λ_*TD*_ yield here a product of the three probability distributions: $P\left(I_{\text{TD}}^{t}|\beta \;\gamma \right)$ from the top-down component, $P\left(I_{\text{BU}}^{t}|S^{t}\;\beta \right)$ from the bottom-up component (which involves sensory processing of the current input frame *S*^*t*^) and $P\left(I^{t}|\left[\lambda_{\text{IJ}}=0\right]X^{t}\right)$ from the memory component.

To conduct our simulations, we first initialize the model for *t* = 0. In Equation 9, this concerns the initial state of the distribution over zones *P*(*J*^0^) in the memory component, which we define as a uniform distribution, and the initial eye position *X*^0^, which we set to the Eyes AOI (as it is both an AOI with high saliency usually, and spatially centered). Then, for every time step *t*, to select the next eye position *X*^*t*+1^, we compute Equation 9, then draw a zone at random according to the computed probability distribution. This can be written as:


(10) $$\begin{array}{l} X^{t+1}\leftarrow \text{draw}\left(P\left(I^{t}|\left[\lambda_{\text{BU}}=1\right]\;\left[\lambda_{\text{TD}}=1\right]\;\left[\lambda_{\text{IJ}}=0\right]X^{t}S^{t}\;\beta \;\gamma \;\theta \right)\right). \end{array}$$

#### 3.2.8 Simulation analyses and comparison with behavioral data

Simulations provide sequences of eye positions, as sequences of the AOIs they land in. The model is too simple to aim at matching behavioral sequences and precisely predict the order and dynamics of AOI exploration. For instance, the model does not contain a precise description of the oculomotor plant and its dynamics, nor is it properly temporally scaled. Therefore, we summarize trajectories by the proportion of time spent on each AOI, and compare the results with experimental data summarized in the same manner. More precisely, as with the experimental data, we use the model to compute the preference score between the Eyes and Mouth regions in the anticipatory window of analysis, for each trial. As a reminder, this measures the ability to anticipate the Non-speech Event (Eyebrow-raise movement) that ends input stimuli.

A simulation therefore provides the evolution of the preference score, for each trial, and for each condition (Lip-protrusion and Eyebrow-raise). We compute a linear regression of these score functions, and retain the start and end points of these regressions. Simulations and behavioral data are compared by computing the mean-squared error (MSE) between the start and end points of the simulated and measured evolution of preference scores.

To explore the ability of the model to account for behavioral data, we perform a grid search on the three-dimensional space defined by the free parameters of the model, that is, β, γ, and θ. We recall that β and γ can be interpreted as respectively controlling the “strength” and “speed” of anticipatory learning in the top-down component, and θ as controlling the weight of static saliency in the bottom-up component. In the grid search, we perform model simulations for all possible combinations of these parameters, with empirically defined value ranges for the three parameters (see Table 2). Thus, for each experimental condition an optimal value of these three parameters is found. We can then compare descriptively the values obtained according to the groups and discuss their meaning and their coherence with the literature.

## 4 Results

In order to test the model, behavioral data in five experimental conditions were considered: bilingual infants of 12, 15, and 18 months, and monolingual infants of 12 and 15 months. In each of these conditions, the objective was to compare the proportion of total looking time (PTLT) to each of the two AOIs: one around the eyes and eyebrows and one around the mouth of the speaker.

The experimental results are presented in three parts. The first part assesses the general preference for the mouth over the eye region of the speaker averaged across all trials, for the whole duration of the Non-Speech and the Speech Events. The second part presents the effect of Non-Speech Event condition (Eyebrow-raise vs. Lip-protrusion condition) for the whole duration of the Non-Speech Event (detection window analysis). The third part of the results presents the effect of Non-Speech Event condition over the course of the experiment (from trial 1 to trial 19), for the last 50% of the Speech Event (anticipatory window analysis).

In these first two sections, only the results for 12-month-old infants will be detailed. Indeed, behavioral results for the 15- and 18-months-old infants are already published in Fort et al. (2018). However, in order to be able to compare all five conditions with each other, the results at 15 and 18 months will be synthesized and a figure will gather all the conditions at the end of each of these two sections. For the ANOVA analyses presented below, three residual plots were observed: the fitted vs. residuals plot, the normal probability plot, and the histogram of residuals. These plots were used to check the assumptions of linearity, normality, and homoscedasticity of the errors. A final section will present the comparison to the model. For this comparison, the model is fitted to the data in order to identify optimal values of the three free parameters for each experimental condition. These values will then be discussed and interpreted.

### 4.1 General preference for the mouth region

#### 4.1.1 Non-speech event

**Figure 4:**
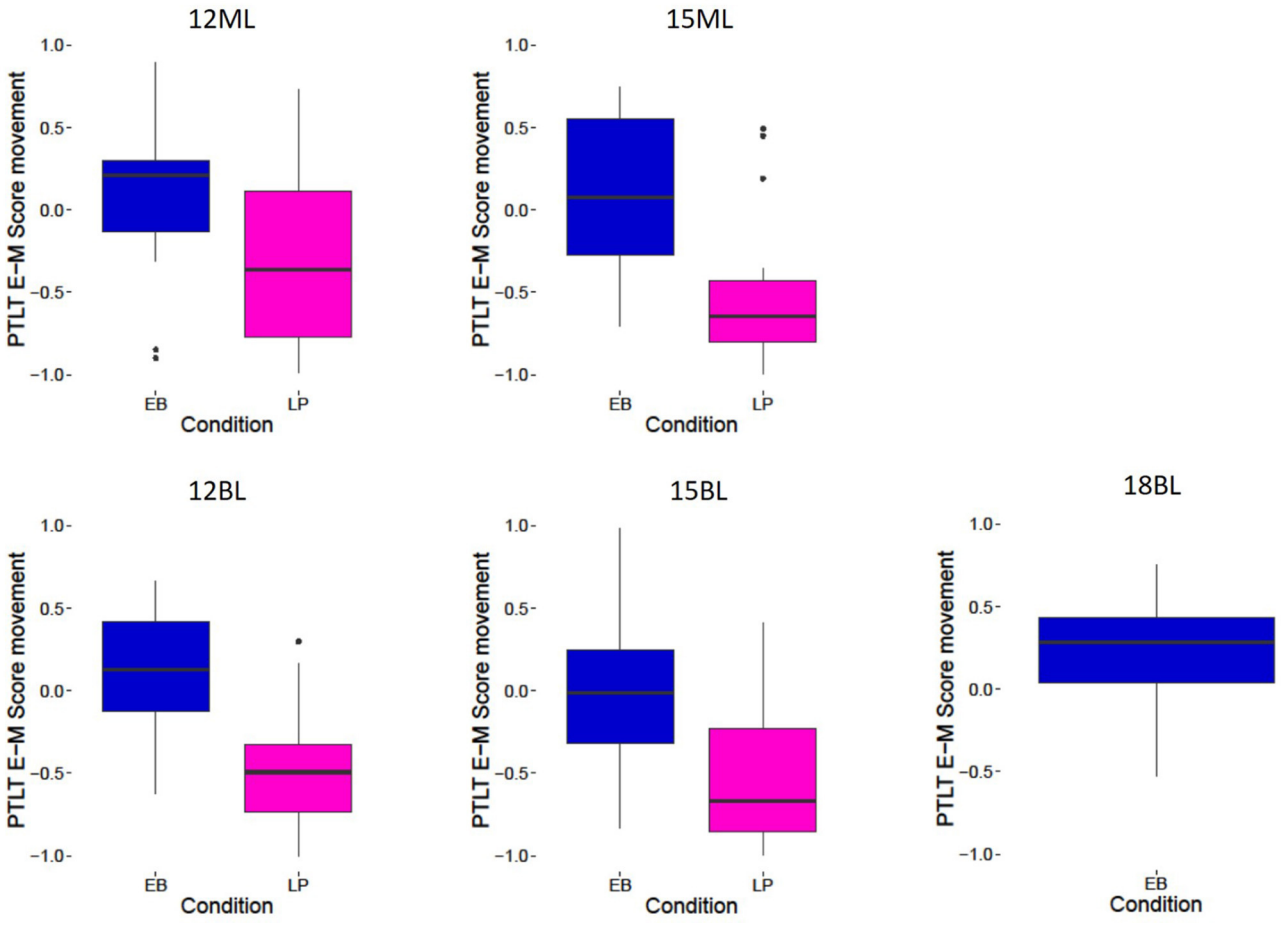
Eyes-Mouth proportion of total looking time (PTLT) score movement, for all conditions (EyeBrow raise EB, Lip Protrusion LP) and all groups (12-month-old monolinguals 12ML, 15-months-old monolinguals 15ML, 12-months-old bilinguals 12BL, 15-months-old bilinguals 15BL, 18-months-old bilinguals 18BL). Positive PTLT values correspond to a preference for the eye region, and negative PTLT values to a preference for the mouth region.

Results for the 12-months-old during the Non-Speech Event are summarized in Figure 4. To assess infants' preference for the speaker's mouth over the eye region during the Non-Speech Event, we first tested whether each infant's Eyes-Mouth PTLT difference scores in this window of analysis significantly differ from zero (i.e., signaling an absence of a preference). Results showed that both bilinguals and monolinguals in the Eyebrow-raise condition looked at both locations of the speaker's face for a similar amount of time (*t*_(19)_ = 1.31, *p* = 0.20 and *t*_(19)_ = 1.14, *p* = 0.27), while in the Lip-protrusion condition, both bilinguals and monolinguals had a strong preference for the mouth region (*t*_(19)_ = −7.28, *p* < 0.001, Hedge's *g* = −7.16 and *t*_(19)_ = −2.12, *p* < 0.05, Hedge's *g* = −2.09, respectively).

#### 4.1.2 Speech event

**Figure 5:**
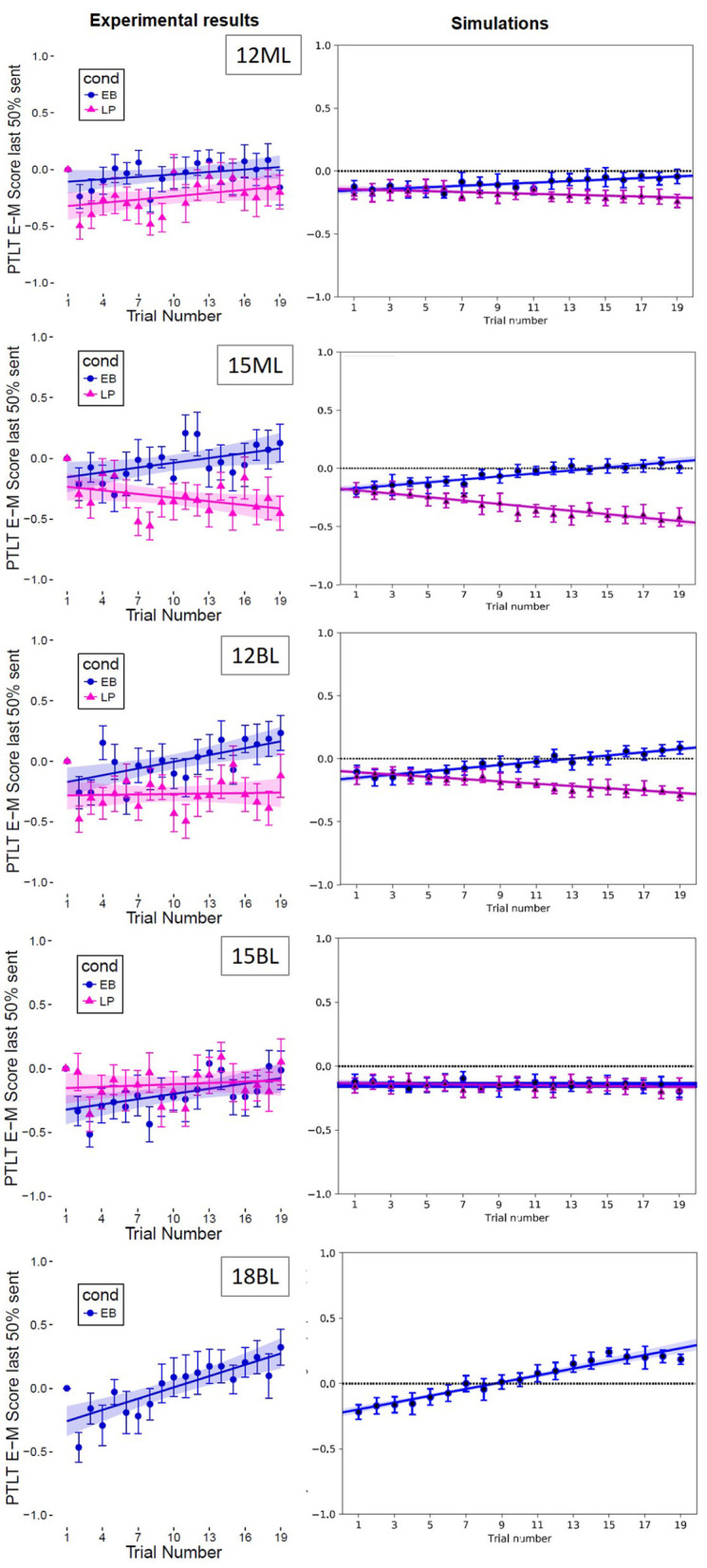
Comparison of experimental **(left column)** and simulation **(right column)** results. Each row refers to an experimental group, with the number indicating the age group (12, 15, and 18-months) and the letter indicating Monolinguals and Bilinguals. Each graph shows the evolution of the Eyes-Mouth PTLT score (*y*-axis) as a function of trial number (*x*-axis) after baseline correction.

Results for the 12-months-old are summarized in Figure 5 left column. As for the detection window of analysis, we first assess infants' general bias for the mouth (over the eyes region) of the speaker on infants' Eyes-Mouth PTLT scores averaged across trials, for the last 50% of the Speech Event. Overall, both bilinguals [Eyebrow-raise condition: *t*_(19)_ = −2.37, *p* < 0.05, Hedge's *g* = −0.73, Lip-protrusion condition: *t*_(19)_ = −7.92, *p* < 0.001; Hedge's *g* = −2.45] and monolinguals [Eyebrow-raise condition, *t*_(19)_ = −4.78, *p* < 0.001; Hedge's *g* = −1.3, Lip-protrusion condition: *t*_(19)_ = −7.08, *p* < 0.001; Hedge's *g* = −2.19] showed a preference for the mouth region during the last 50% of the Speech Event. See Supplementary material (Appendix S3) for further analyses showing that PTLT scores to the Eyes or Mouth AOIs are representative of most infant gaze behavior (i.e., 72 to 85%) for the whole duration of the experiment.

### 4.2 Detection window of analysis

We then explored whether bilinguals and monolinguals could detect the Eyebrow-raise or the Lip-protrusion movement, by submitting these Eyes-Mouth PTLT scores averaged across trials and across the whole duration of the Non-speech Event (detection window) to a 2 (Language group: Bilinguals, Monolinguals) × 2 (Non-Speech Event type: Eyebrow-raise or Lip-protrusion) between-participants ANOVA (AIC: 104, BIC: 116). The main effect of Non-Speech Event type was significant [*F*_(1,76)_ = 23.1, *p* < 0.001, Hedge's *g* = 1.1]. However, neither the main effect of Language Group [*F*_(1,76)_ = 1.22, *p* = 0.27] nor the interaction between the two factors [*F*_(1,76)_ = 1.24, *p* = 0.27] were significant. Thus, these results show that in spite of their predictable general bias for the mouth region of the speaker, both bilinguals [*t*_(38)_ = 5.31, *p* < 0.001, Hedge's *g* = 1.6] and monolinguals [*t*_(38)_ = 2.25, *p* < 0.05, Hedge's *g* = 0.7] could detect the Eyebrow-raise and the Lip-protrusion Non-Speech Event during their presentation. These results are similar to the ones observed in 15-months-old and 18-months-old infants (Fort et al., 2018). At all ages and for all language groups, infants could successfully detect the Non-Speech Event.

### 4.3 Anticipation window of analysis

To track the time course of infants' looking behaviors over the course of the experiment, we then considered their Eyes-Mouth PTLT preference scores for both bilinguals and monolinguals at each trial (from trial 1 to trial 19, see Figure 5). As in Fort et al. (2018), we baseline corrected the preference scores for each participant from trial 2 to trial 19. To do so, we used the initial preference for the eyes or the mouth region of the speaker at trial 1 (when they still had no exposure to the Non-Speech Event) as a reference, as described in the formula:


(11) $$\{\begin{array}{ll} EM_{bc}t_{n}=\frac{EMt_{n}-EMt_{1}}{1-EMt_{1}} & \text{if}\;{\text{EMt}}_{\text{n}}>{\text{EMt}}_{\text{1}},\\ EM_{bc}t_{n}=\frac{EMt_{n}-EMt_{1}}{1+EMt_{1}} & \text{otherwise,} \end{array}$$

where *EM*_*bc*_*t*_*n*_ represents the baseline corrected Eyes-Mouth PTLT, and *EMt*_1_ and *EMt*_*n*_ the Eyes-Mouth PTLTs at trial 1 and trial *n*, respectively. The time course of the baseline corrected Eyes-Mouth PTLTs averaged across participants are presented in Figure 5; a positive score indicates an increased preference for the eyes area and a negative one an increased preference for the mouth area.

We then applied Growth Curve Analysis (Mirman, 2017) to these baseline corrected Eyes-Mouth PTLT preference scores. Analyses were carried out in R version 3.0.2 (Team, 2013) using the lme4 package (version 1.0-5, Bates et al., 2012). The overall learning curves, for each condition, were linearly fitted with fixed effects of Non-Speech Event (Eyebrow-raise, Lip-protrusion) on all time terms (i.e., Trial Number). The Lip-protrusion condition was treated as baseline and parameters were estimated for the Eyebrow-raise condition. The model also included random effects of participants on all time terms. The fixed effects of Non-Speech Event were added individually and their effects on model fit were evaluated using model comparisons. Improvements in model fit were evaluated using –2 times the change in log-likelihood, which is distributed as χ^2^ with degrees of freedom equal to the number of parameters added. Table 3 shows the fixed effect parameter estimates and their standard errors along with p-values estimated using Satterthwaite's approximation for degrees of freedom for the last 50% of the Speech Event.

**Table 3:** Fixed effects of growth learning curve model.

	**Estimate**	**Std. Error**	**df**	***t* value**	**Pr(>∣*t*∣)**
**12 month-old bilinguals:**					
Intercept	–0.274865	0.078798	40.290446	–3.488	0.00119^**^
Trial number	0.001646	0.006106	38.698913	0.270	0.78893
Cond. ER	0.100692	0.111499	40.392008	0.903	0.37184
Trial: Cond.ER	0.017574	0.008633	38.823393	2.036	0.04864^*^
**12 month-old monolinguals:**					
Intercept	–0.319017	0.060839	42.242415	–5.244	4.74e-06^***^
Trial number	0.008883	0.004384	39.263835	2.026	0.0496^*^
Cond. ER	0.209976	0.083996	41.124400	2.500	0.0165^*^

Cond. ER, Condition Eyebrows raise; Trial: Cond.ER, Trial Number for Condition Eyebrows raise. ^*^p < 0.05, ^**^p < 0.01, ^***^p < 0.001.

For the bilinguals, the effect of the Non-speech Event on the intercept already improved the model fit on its own (χ^2^(1) = 4.01, *p* < 0.05, AIC: 1212, BIC: 1244) as well as significantly improving it on the Trial Number term (χ^2^(1) = 3.94, *p* < 0.05, AIC: 1210, BIC: 1246). For the monolinguals, the effect of the Non-Speech Event on the intercept improved the model fit on its own (χ^2^(1) = 5.82, *p* < 0.05, AIC: 1072, BIC: 1105) but did not significantly improve it on the Trial Number term (χ^2^ < 0.1). Thus, these results show that, for both the monolingual and the bilingual groups, infants in the Eyebrow-raise condition, as compared to the infants in the Lip-protrusion condition, increased their looking time at the eye region of the speaker. However, the effect remained stable across trials for the monolinguals, while it increased over the course of the experiment for bilinguals. This means that at 12 months of age, infants were able to disengage their focus of attention from the talking mouth of the speaker to anticipate the appearance of the Eyebrow-raise movement in the eye region. However, this learning curve was robust for the bilinguals, but somewhat weaker for the monolinguals.

Interestingly, the results for the anticipation window of analysis at 12 months differ from the ones previously obtained at 15 and 18 months (Fort et al., 2018). For the monolingual groups, the pattern of results is quite straightforward. The anticipation was weak for the younger 12-month-old monolingual group, and improved for the group of 15-months-old monolinguals. This suggests that their performance improved with attentional control and/or language maturation. In the bilingual groups of infants however, a U-shaped developmental pattern was observed. While the bilingual groups were clearly able to anticipate the Eyebrow-raise movement at 12 months and at 18 months, the bilinguals at 15 months did not anticipate the Eyebrow-raise movement. This last point will be further addressed in the General Discussion.

### 4.4 Adjustment of the free parameters and comparison with the model

In order to compare the model with experimental data, we first defined a set of possible values for each parameter. In a second step, we tested the adequacy between the simulated and experimental results for each combination of parameters, applying a classical grid search method.

#### 4.4.1 Exploratory analysis

**Figure 6:**
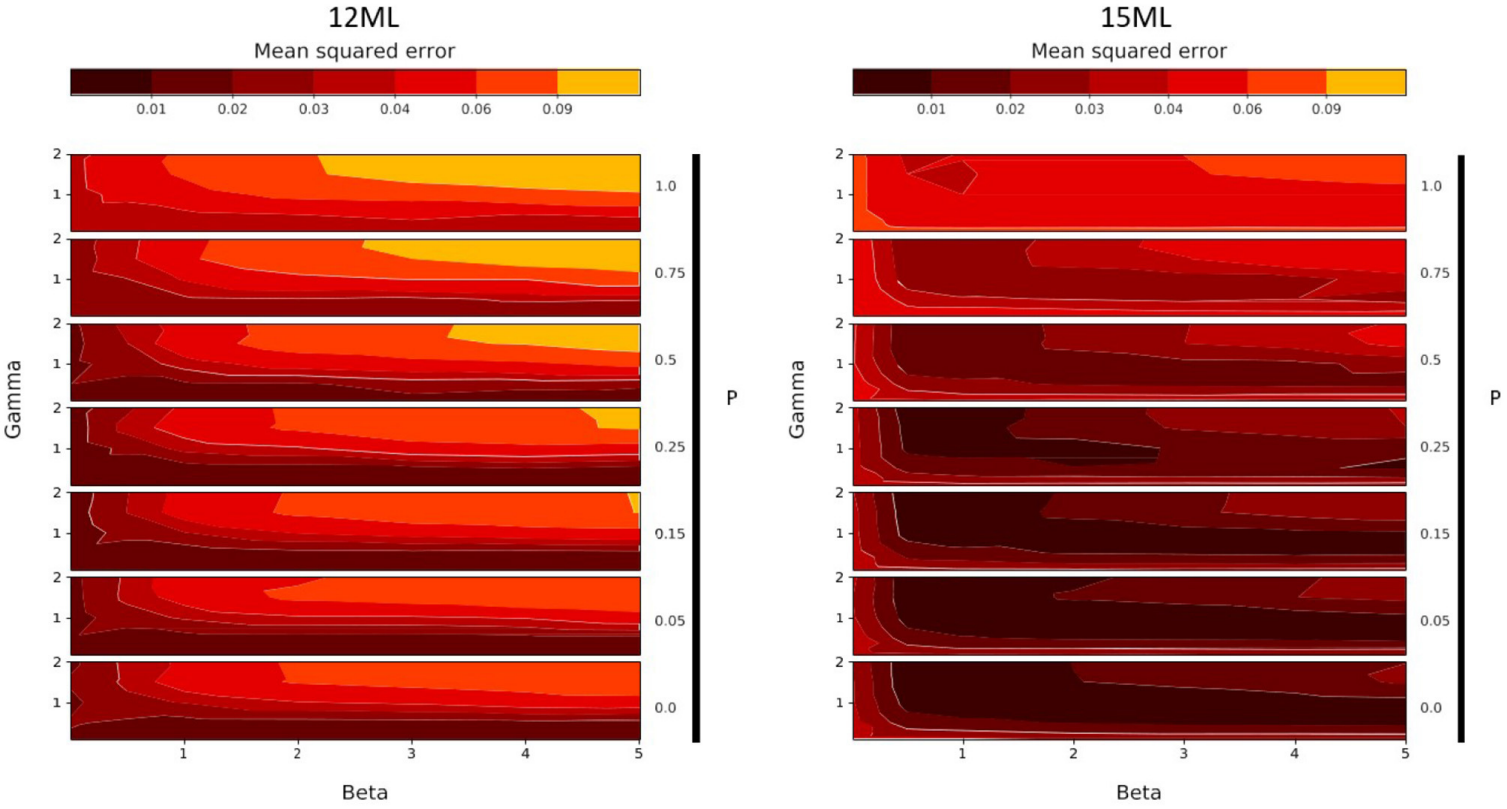
Grid search results, over parameters β (noted Beta), γ (noted Gamma) and θ (noted Theta), for 12-months-old monolinguals (12ML, **left**) and 15-months-old monolinguals (15ML, **right**). Each plot represents the model fit measure (MSE) with a color gradient, from black (good fit) to orange (poor fit).

**Figure 7:**
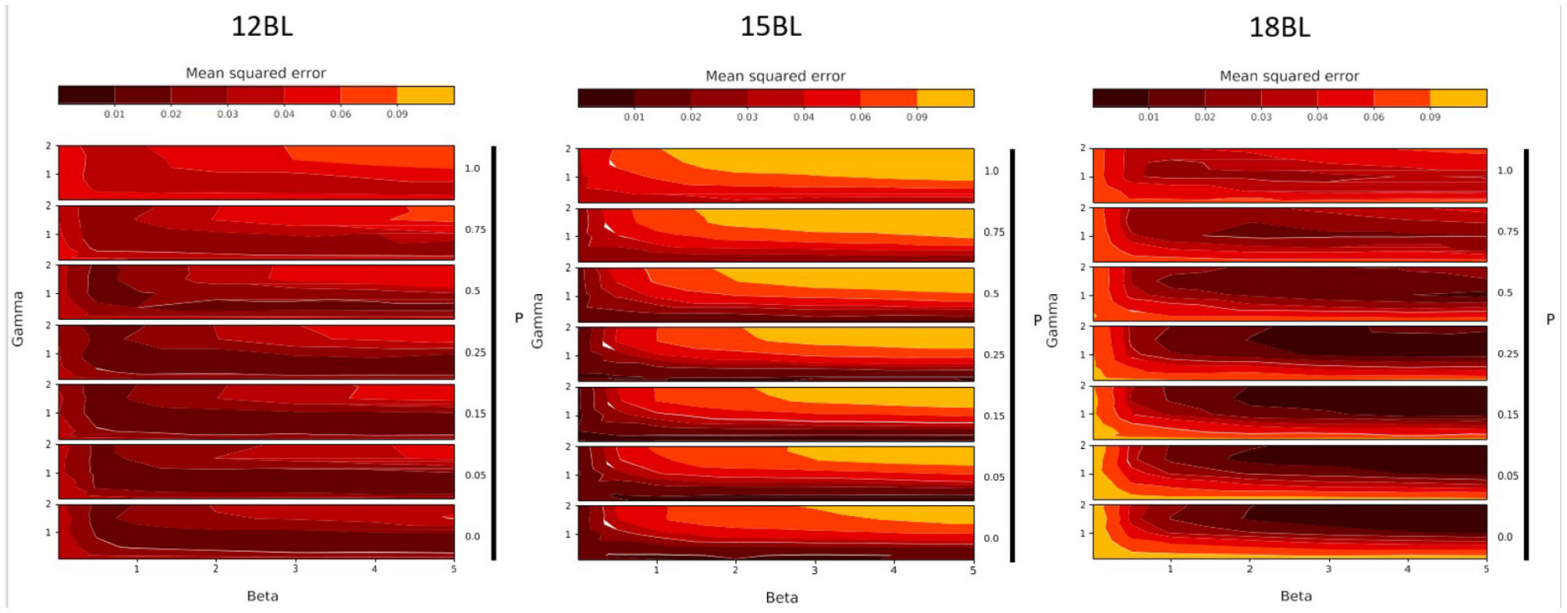
Grid search results, over parameters β (noted Beta), γ (noted Gamma) and θ (noted Theta), for 12-months-old bilinguals (12BL, **left**), 15-months-old bilinguals (15BL, **middle**) and 18-months-old bilinguals (18BL, **right**). Each plot represents the model fit measure (MSE) with a color gradient, from black (good fit) to orange (poor fit).

In order to estimate the optimal values for each free parameter of the model, we conducted this exploratory analysis in each experimental condition. The results are presented for the monolingual groups in Figure 6 and for the bilingual groups in Figure 7.

These two figures describe the evolution of the fit of the simulated results to the experimental results according to different values of the three free parameters. On each subplot, the x-axis corresponds to the variation of the parameter β (strength of Top-Down learning), while the y-axis corresponds to the different values of γ (speed of anticipation). The variation of the parameter θ (weight of the static salience, noted Theta in the Figures) is represented by the vertical stacking of subplots, indicated by the arrows. Concerning this last parameter, the closer θ is to 1, the greater the importance of static salience, which translates to a preference for the eyes. Conversely the closer θ is to 0, the greater the importance of movement, that is, a preference for the mouth.

The results of this grid search exploration provide us with several observations. First of all, they allow us to test certain qualities of the model. Indeed, we observe that, for each group, a variation of the input parameter values leads to a variation of the fit of the model to the output experimental data. The predictions of the model are neither perfectly accurate everywhere, nor inaccurate everywhere, but they do account for the experimental results at some points of the parameter space, while this is less true at other points. The model thus has a first quality: sensitivity. A second important point is that these good parameter values are not isolated. We do not observe a particular good fit for a small region of the parameter space and a bad fit otherwise, nor the opposite. Therefore, the model is robust. Finally, we do not observe that good fit regions are scattered randomly in the parameter space. Instead, we notice very easily identifiable and distinct trends between groups. Therefore, the model is smooth.

#### 4.4.2 Optimal value of free parameters and interpretation of the model

**Figure 8:**
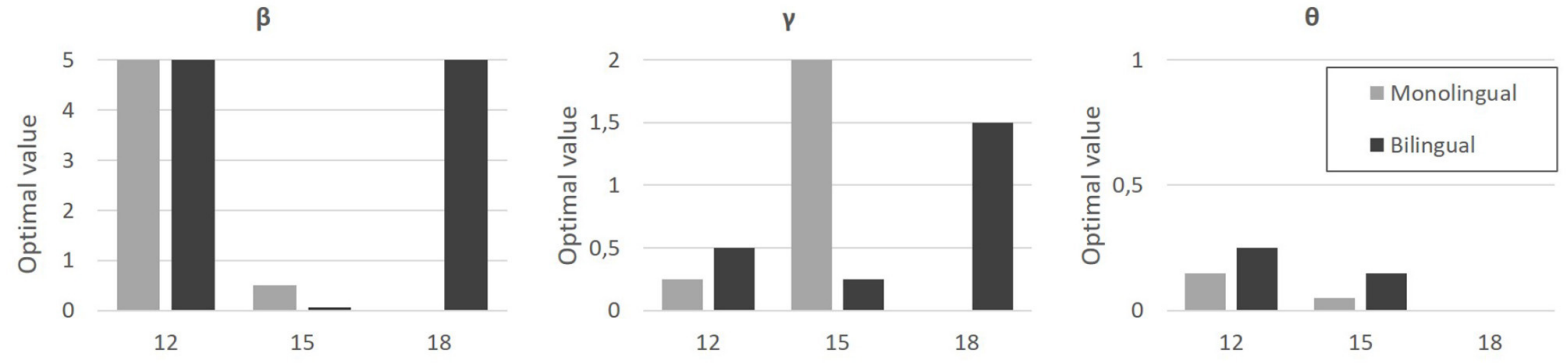
Optimal values (*y*-axis) found for parameter β **(left)**, γ **(middle)**, and θ (**right** plot), for each age group (*x*-axis, 12, 15, and 18-months-old) and for monolingual (light gray bars) and bilinguals (dark gray bars).

The exploratory analysis performed on the parameter space revealed, for each group, optimal values for each parameter (e.g., the combination of parameters that minimizes the mean squared error). These values are given in Figure 8.

The θ parameter, associated with the bottom-up component, weights the static saliency map in the model. The optimal θ values seem to indicate a general increased preference for dynamic areas, and this effect seems to strengthen with age. It also appears that this preference is less marked for bilinguals, and increases later to be total (θ = 0) at 18 months, whereas it is already close to 0 for 15-months-old monolinguals. This movement saliency corresponds to a preference for the mouth, a preference that is also found in the experimental data. All this attests to the consistency of the model with the experimental data.

The β parameter, associated with the top-down component, controls the “strength” of anticipatory learning in the model. The values seem to indicate maximum anticipation at 12 and 18 months (β = 5), with almost no anticipation at 15 months, and no observable difference between monolinguals and bilinguals.

The γ parameter, also associated with the top-down component, controls for the “speed” of anticipatory learning in the model. The values seem to indicate an increase in the speed of anticipatory learning with age, with a difference related to bilingualism. Indeed, the increase in speed seems to take place between 12 and 15 months for monolinguals, and between 15 and 18 months for bilinguals.

#### 4.4.3 Adequacy between the simulated results and the experimental results

A comparison was then made between the experimental results and the simulations, based on optimal parameter values; it is shown in Figure 5. It is another way of visualizing the fit between simulations and experimental data for optimal points in the parameter space for each group. This shows that the model is capable of simulating the same trends as observed in the experimental results.

## 5 Discussion

In this paper we presented a computational model predicting how infants distribute their attention to talking faces. The model was fitted and confronted to eye-tracking data from 12- to 18-month-old infants when exploring talking faces providing information in the eyes or the mouth region that changes over time. The data for the 15- and 18-months-old were already available (Fort et al., 2018), and we added new, original data on 12-months-old monolingual and bilingual infants.

### 5.1 Discussion of experimental results

#### 5.1.1 A general preference for the talking mouth during the second year

The first result that arises from the present experimental eye-tracking data is that in the beginning of their second year (12–18 months), infants still show, at the group level, a strong bias for the talking mouth (over the eyes) region of the speaker. This is in line with other findings, suggesting that the peak of mouth preference happens at around 15–18 months of age (e.g., de Boisferon et al., 2018; Morin-Lessard et al., 2019; Sekiyama et al., 2021). If this preference is indeed related to language learning (as it is modulated by language familiarity in the first and second year, e.g., Lewkowicz and Hansen-Tift, 2012; Pons et al., 2015; Morin-Lessard et al., 2019), a number of processes could explain why infants still need to focus on the mouth region of faces speaking in their native language(s). We detail below two potential (non-mutually exclusive) language learning mechanisms that could explain this behavior and that are supported by findings in the literature.

The first potential language learning mechanism concerns the significant infant vocabulary growth that usually begins during the second year (Vihman, 1996). This stage of development is usually characterized by a dramatic improvement in their lexical knowledge and word production skills. While this “vocabulary burst” has been widely reported in the literature, the mechanisms underlying this phenomenon remain unclear (Bergelson, 2020). Focusing on a talking mouth gives direct access to redundant audiovisual cues, which could help the processing and acquiring of phonological representations of novel word forms (de Boisferon et al., 2018; Fort et al., 2018), which could, in turn, enhance word retention and memory and further vocal imitations and word production attempts (see Introduction and (Young et al., 2009; Tenenbaum et al., 2015; Tsang et al., 2018), for data in support of this hypothesis).

The second potential language learning mechanism concerns the acquisition of morphosyntactic non-adacent rules, a key component of syntactic processing (Santelmann and Jusczyk, 1998; Gómez and Maye, 2005). For instance, infants learn non-adjacent dependencies when hearing spoken sentences such as “mommy is [work]ing” or “grandma is [look]ing.” To track and memorize the statistical regularity between “is” and “-ing,” infants need to learn the non-adjacent temporally distant relationship between these two key elements, while ignoring intermediate ones such as [work] and [look]. While infants seem to be able to track these statistical regularities early on (see Rabagliati et al., 2019, for a meta-analysis), this ability remains quite weak until the second year (Marchetto and Bonatti, 2015). Focusing on the articulatory information provided by the talking mouth could help sustain their attention on the speech stream long enough to enhance their ability to track, extract and memorize the non-adjacent syntactic rules of their native language(s). Indeed, temporal attention is considered crucial for this type of learning (de Diego-Balaguer et al., 2016). While the causal relationship between mouth looking and syntactic rule learning remains to be proven, it is worth mentioning that in line with this hypothesis, a recent study (Birulés et al., 2022) showed that 15-month-old infants increased their attention to the mouth of a talking face producing novel non-adjacent syntactic rules, compared to when they were presented with a face producing familiar ones.

#### 5.1.2 Comparing the developmental trajectories between monolingual and bilingual infants

The second experimental result that arises from the present data is that monolingual and bilingual infants exhibit different developmental trajectories when looking at talking faces. For monolinguals, results show that in spite of an initial preference for the mouth region of the face speaking in their native language, they could disengage from it to look at the eye region and anticipate the apparition of the eyebrow-raise movement. This ability seems to improve with age: the anticipation was weak at 12 months, but clear at 15 months. These results could be interpreted as a maturation and improvement of top-down attentional control, or by the fact that 15-month-old monolinguals needed the information provided by the mouth region of the speaker less than 12-months-old. Contrary to monolinguals, the bilinguals' ability to disengage from the talking mouth in the eyebrow-raise condition did not follow a linear but a U-shaped developmental pattern. While they clearly could anticipate the eyebrow-raise movement at 12 and 18 months of age, they did not show any sign of anticipation at 15 months. This suggests that at this age, they prioritized the information provided by the talking mouth of the speaker over the information provided by the eye region during the course of the whole experiment (for reviews on attentional control and bilingualism, see Costa et al., 2008; Woumans et al., 2015; D'Souza and D'Souza, 2021; Privitera et al., 2023). Non-linear U-shaped (or inverted U-shaped) developmental patterns are commonly observed in infancy, and especially in bilingual infants' language learning trajectories (e.g., Bosch and Sebastián-Gallés, 2003; Burns et al., 2007; Sebastián-Gallés and Bosch, 2009; Garcia-Sierra et al., 2011). Importantly, large differences are found between bilingual and monolingual infants when processing phonological word forms during word learning or word recognition (e.g., Fennell et al., 2007; Havy et al., 2016; Höhle et al., 2020; for a review, see Höhle et al., 2020) which could potentially explain the different attentional strategies to talking faces in the present study. Indeed, one of the language milestones at 15 months cited above is the onset of vocabulary burst and word production. The increased reliance on the mouth movement of the speaker by the bilingual infants could be due to the fact that Catalan and Spanish are close languages (Bosch and Sebastián-Gallés, 2003; Sebastián-Gallés and Bosch, 2009). Notably, Catalan and Spanish share a significant number of cognates that are close semantically and phonologically. Such small phonological distance between each lexical unit without any other information to distinguish them (e.g., semantic or syntactic) may compel bilinguals to center their attention on the articulatory gestures of the speaker to avoid confusing them, regardless of the task at hand, during the whole course of the experiment. Accordingly, this increased reliance on the mouth region of talking faces has also been found in previous studies testing other Catalan-Spanish bilingual infants (Pons et al., 2015; Ayneto and Sebastian-Galles, 2017; Birulés et al., 2019), but not for more phonologically and semantically distant bilinguals: Spanish-Basque (Pejovic et al., 2021), French-English (Morin-Lessard et al., 2019). It is important to note however that this interpretation remains entirely speculative, and other language learning processes might explain the different pattern of results between monolinguals and bilinguals.

As a conclusion, several potential language learning mechanisms could be at the origin of the general mouth preference and explain the difference in behaviors between monolingual and bilingual infants during the second year of development. While additional experimental studies are required to establish whether and to what extent attention control to the mouth is linked with different language learning processes, an experimental approach alone can only provide correlational evidence between the two behaviors. A complementary approach such as mathematical modeling is required to get closer to causal mechanistic explanations of language learning in infancy. Indeed, experimental data alone does not allow us to extract exact relative weights of the bottom-up and top-down components in infant behavior, nor to generalize these predictions to large sets of constraints and situations. In contrast, we used computational modeling and simulations to implement a theoretical architecture of attentional control maturation (e.g., by varying the weight of the top-down attention component) and compare the results of model simulations to word-learning experimental data. In the following section, we discuss the conclusions that can fairly be drawn from the PROTOBOT computational model and the presented simulations.

### 5.2 Discussion of the comparison of the model simulations with the experimental data

#### 5.2.1 What conclusions can be drawn from the model?

The PROTOBOT model of visual attention allocation we presented in this study combines three main components, each with a dedicated scope. It uses Bayesian inference to simulate infant gaze trajectories on a visual scene displaying a talking face. We then constrained the model to simulate the task of the experimental conditions of Fort et al. (2018), before performing a number of simulations for varying parameter values. Comparing the simulated results to our experimental results, we found that the model is able, for optimal parameter values, to faithfully reproduce the results of the different groups of infants tested.

The first conclusion we can draw concerns the model itself, which, to the best of our knowledge, is the first probabilistic model of visual attention that teases apart, explicitly represents, and combines the three main components (bottom-up, memory, and top-down) assumed in theoretical accounts of visual attention. Even though the model was somewhat under-specified, in the sense that it remains at this stage agnostic with respect to precise sensory and learning mechanisms, our study suggests that it is already capable of capturing trends in visual attention orientation. More precisely, our comparison with experimental data showed that its three parameters effectively modulate the simulated visuo-attentional behavior: as θ varies, the model is more or less reliant on static or dynamic visual cues; as β and γ vary, the model anticipates more or less strongly, and more or less early, a visual event outside of the most visually relevant AOI. We also showed that the chosen parameter space for the model made it capable of capturing a range of varied behavior in a smooth and robust manner, allowing us to compare experimental observations and identify adequate parameter values in a sensitive fashion. These structural properties of the model suggest that it is a sound and, we hope, useful tool for visuo-attentional behavior analysis.

Moreover, the simulated behaviors are consistent with the experimental results presented in this study and those of the previous study (Fort et al., 2018). In particular, we can consider the increase in anticipation speed with age, which occurs later for bilinguals, as faithfully captured by the model. Similarly, the simulated results show a preference for motion saliency in all groups, in agreement with the literature (Wolfe and Horowitz, 2004). Finally, our results show that the 18-month-old bilingual group is the most successful in achieving the expected anticipation compared to the other groups tested. The model was also able to capture the decline in the ability to anticipate for 15-month-olds, for both monolinguals and bilinguals, with the β parameter values.

#### 5.2.2 Limitations and perspectives

If we return to the questions posed in the Introduction, and to the hypothesis we aimed to test, we must note that the proposed PROTOBOT model has descriptive power, in the sense that it can account for observed experimental data, but relatively limited explanatory power. It should be remembered that our main theoretical aim was to explain why the 15-month-old bilinguals focused their visual attention on the mouth more than their monolingual counterparts. While the model reproduces this tendency well, notably thanks to its parameter controlling the evolution of, and reliance on, top-down knowledge, it provides little evidence to explain how such parameters would vary.

Indeed, at this stage, the PROTOBOT model only features a “surface description” of the learning mechanisms involved in the maturation of task-related knowledge that influence visuo-attentional control in a top-down manner. A more detailed, explanatory model would describe mechanisms for understanding words and sentences, learning new words and incorporating them in a known lexicon. The attentional requirements of these processes would then depend on the current state of the lexicon, on whether it involves a single lexicon for monolinguals or two lexicons for bilinguals, and therefore on the difficulty of phonological processing.

More precisely, the model could be extended to implement phonological processing and lexical acquisition (Feldman et al., 2009, 2013). Such a model could help explore the cognate hypothesis developed by (Bosch and Sebastián-Gallés, 2003). Consider for instance Spanish-Catalan bilinguals, for which, during lexical acquisition, there is higher uncertainty on vocalic phonetic learning of categorical boundaries. Indeed, whereas semantic and syntactic cues also help monolinguals build their phonological representations of the lexicon, due to the presence of semantically and syntactically close cognates, bilinguals face a more difficult learning situation. Our model, marrying eye-trajectory prediction with phonological mechanisms from Feldman et al. (2009, 2013), could test whether a greater reliance on the mouth region would be a strategy, for bilinguals, to counteract this phonological uncertainty (see Section Comparing the developmental trajectories between monolingual and bilingual infants).

Such a more detailed model could help assess whether the theoretical frameworks proposed in the literature, linking phonological processing demands to visuo-attentional behaviors, adequately explain the variety of experimental observations, in mono- vs bilinguals, and across age groups. Such developments are, of course, topics for future studies.

Finally, in current works, and to study the model's generalizability, we have reprised and adapted the model to another, quite different experimental situation. We have asked participants to play a video game with two subtasks: first, they had to move a cursor to avoid falling balls following trajectories that were more or less easy to anticipate; second, they had to simultaneously monitor and control a continuously emptying gauge to be at a desired level. The PROTOBOT model was adapted to this situation in order to predict the participants' oculomotor behavior according to predefined zones relevant to both subtasks. The major difference with the present study is the introduction of a second task, implying the introduction of the notion of task priority into the model. Taking into account the multitasking aspect in the model would enable to apply it to more ecological situations such as automobile driving, where relevant zones can be clearly defined for the different subtasks in progress.

## Data availability statement

The raw data supporting the conclusions of this article will be made available by the authors, without undue reservation.

## Ethics statement

The studies involving humans were approved by Center for Brain and Cognition, Universitat Pompeu Fabra, Barcelone, Espagne. The studies were conducted in accordance with the local legislation and institutional requirements. Written informed consent for participation in this study was provided by the participants' legal guardians/next of kin. Written informed consent was obtained from the individual(s), and minor(s)' legal guardian/next of kin, for the publication of any potentially identifiable images or data included in this article. Written informed consent was obtained from the individual(s) for the publication of any identifiable images or data included in this article.

## Author contributions

SL: Conceptualization, Project administration, Supervision, Validation, Visualization, Writing – original draft, Writing – review & editing. BF: Formal analysis, Investigation, Methodology, Visualization, Writing – original draft, Writing – review & editing. NS-G: Conceptualization, Data curation, Funding acquisition, Project administration, Supervision, Writing – review & editing. RB: Conceptualization, Writing – review & editing. JD: Conceptualization, Data curation, Formal analysis, Funding acquisition, Methodology, Project administration, Supervision, Validation, Visualization, Writing – original draft, Writing – review & editing. MF: Conceptualization, Data curation, Formal analysis, Funding acquisition, Investigation, Methodology, Project administration, Supervision, Validation, Visualization, Writing – original draft, Writing – review & editing.

## Footnotes

^1^We use the Python implementation available at https://docs.opencv.org/3.4.0/da/dd0/classcv_1_1saliency_1_1StaticSaliencyFineGrained.html.
