# Supplementary material for: Monolingual and bilingual infants' attention to talking faces: evidence from eye-tracking and Bayesian modeling
Source: Front Psychol. 2024 Mar 14;15:1373191. doi: 10.3389/fpsyg.2024.1373191 (PMC10973108; doi:10.3389/fpsyg.2024.1373191)
Supplement: Supplementary file 1 [file Presentation_1.pdf]

Supplementary Material

S1 Appendix.

Inversion mechanism based on coherence variables.

To define an inversion mechanism using coherence variables, we rely on their original definition (Gilet et al., 2011) as “Bayesian switches”, which we recall here. In the memory component, coherence variable λ_{IJ} links variables I^t and J^t by probability distribution $P(\lambda_{IJ} \mid I^t\ J^t)$, so that, with the usual definition of coherence variables, we have:

$$P([\lambda_{IJ} = 1] \mid [I^t = i^t]\ [J^t = j^t]) = \begin{cases} 1 & \text{if } i^t = j^t \\ 0 & \text{otherwise .} \end{cases} \tag{S1}$$

Assume that this probability distribution is accompanied by an arbitrarily defined distribution $P(J^t)$ on the one hand, and a uniform distribution $P(I^t)$ on the other hand. Consider computing $P(I^t \mid [\lambda_{IJ} = 0])$. Bayesian inference yields:

$$\begin{aligned} P([I^t = i^t] \mid [\lambda_{IJ} = 0]) &\propto \sum_{J^t} P(I^t\ J^t\ [\lambda_{IJ} = 0]) \\ &\propto \sum_{J^t} P(I^t) P(J^t) P([\lambda_{IJ} = 0] \mid I^t\ J^t) \\ &\propto \sum_{J^t \neq i^t} P(J^t) \\ &\propto 1 - P([J^t = i^t])\ , \end{aligned} \tag{S2}$$

with $\propto$ the proportional relationship, that is, indicating equality up to a proportional constant. The proportional constant can be computed as the renormalization constant of probability distribution $P([I^t = i^t] \mid [\lambda_{IJ} = 0])$:

$$\begin{aligned} \sum_{I^t} P([I^t = i^t] \mid [\lambda_{IJ} = 0]) &= \sum_{I^t} 1 - P([J^t = i^t]) \\ &= \sum_{I^t} 1 - \sum_{I^t} P([J^t = i^t]) \\ &= z - 1\ , \end{aligned} \tag{S3}$$

with z the cardinal of the domain of variables I^t and J^t. Therefore, the final expression is:

$$P([I^t = i^t] \mid [\lambda_{IJ} = 0]) = \frac{1 - P([J^t = i^t])}{z - 1}\ . \tag{S4}$$

This demonstrates our desired result, and shows how assuming a coherence variable to be 0 provides an “inversion mechanism” for probability distributions.

S2 Appendix.

Complete Bayesian inference.

Here, we derive the main simulation equation of the model, Eq. (9), which we recall here:

$$\begin{aligned} & P(I^t \mid [\lambda_{BU}=1]\,[\lambda_{TD}=1]\,[\lambda_{IJ}=0]\;X^t\;S^t\;\beta\;\gamma\;\theta) \\ \propto\;\; & P(I^t_{TD} \mid \beta\;\gamma)P(I^t_{BU} \mid S^t\;\beta)\left[1-\frac{P(X^t \mid J^t)}{P(X^t)}\sum_{J^{t-1}} P(J^t \mid J^{t-1})P(J^{t-1})\right]. \end{aligned} \tag{S5}$$

The first derivation step to compute :

$$P(I^t \mid [\lambda_{BU}=1]\,[\lambda_{TD}=1]\,[\lambda_{IJ}=0]\;X^t\;S^t\;\beta\;\gamma\;\theta), \tag{S6}$$

relies on the "separation properties" of coherence variables as Bayesian switches. Since both λ_{BU} and λ_{TD} are connected to variable I^t, setting them to 1 implies that the probability distribution over I^t is "connected" to those over I^t_{TD} and I^t_{BU}, and the final result is the product of the three distributions. In other words, computations can be considered "locally" in each component, aiming at probability distributions over variables I^t, I^t_{TD} and I^t_{BU} respectively, and using any available evidence in each component.

Therefore, we now consider $P(I^t_{TD} \mid \beta\;\gamma)$ in the top-down component, $P(I^t_{BU} \mid S^t\;\beta)$ in the bottom-up component, and $P(I^t \mid [\lambda_{IJ}=0]\;X^t)$ in the memory component. The first two of these are trivially computed, since they appear as is in Eq. (7), the decomposition of the joint probability distribution that defines the model.

We now consider computing $P(I^t \mid [\lambda_{IJ}=0]\;X^t)$, locally in the memory component. We first apply the inversion mechanism, then Bayes' theorem and the marginalization rule, to obtain:

$$\begin{aligned} P(I^t \mid [\lambda_{IJ}=0]\;X^t) &= \frac{1}{z-1}\left[1-P(J^t \mid X^t)\right] \\ &= \frac{1}{z-1}\left[1-\frac{P(X^t \mid J^t)}{P(X^t)}P(J^t)\right] \\ &= \frac{1}{z-1}\left[1-\frac{P(X^t \mid J^t)}{P(X^t)}\sum_{J^{t-1}} P(J^t\;J^{t-1})\right] \\ P(I^t \mid [\lambda_{IJ}=0]\;X^t) &= \frac{1}{z-1}\left[1-\frac{P(X^t \mid J^t)}{P(X^t)}\sum_{J^{t-1}} P(J^t \mid J^{t-1})P(J^{t-1})\right]. \end{aligned} \tag{S7}$$

This is the desired result, and concludes the derivation for Eq. (9).

S3 Appendix.

Gaze distribution in the four AOI.

To investigate whether the restricted analyses on the PTLTs to the Eyes and Mouth of the speaker reflect most of infants' gaze behavior, we conducted additional analyses considering infants' total looking times, that is, including the "Other" AOI in our analyses, reflecting times when infants were looking away from the Face and/or the screen. To do so we computed the proportion of total looking time (PTLT) to each of

the four AOI for each participant by dividing the total looking time (TLT) at each AOI (e.g., TLT Eyes) by the total looking time at the four AOIs (TLT Eyes + TLT Mouth + TLT Rest of Face + TLT Other). We then applied a mixed-effects model to analyze these PTLTs scores, for the last 50% of the Speech Event using R and the `lme4` package (alpha level: 0.05, two-tailed). The AOI factor (Eyes, Mouth, Rest of Face and Other) was declared as a within-participant fixed factors. The Other AOI was used as intercept. Each analysis was conducted separately for each age x language x condition group.

12-month-old bilinguals

For the 12-month-old bilinguals in the Eyebrow-raise condition, results (AIC: 8090; BIC: 8132) revealed that infants looked significantly longer at the Eyes ($PTLT_{Eyestotal} = 27.7\%, SD = 6.2\%; Estimate = .98, SE = .22, t = 4.7, p < .001$) or at the Mouth AOIs ($PTLT_{Mouthtotal} = 45\%, SD = 10.1\%; Estimate = 2.8, SE = .22, t = 12.7, p < .001$) than at the Other AOI ($PTLT_{Other} = 18\%, SD = 10.1\%$). They however looked less time at the Rest of the Face AOI ($PTLT_{ROFtotal} = 9.3\%, SD = 3.3\%; Estimate = -.35, SE = .17, t = -2.1, p < .05$) than at the Other AOI. In other words, these results show that the analyses presented in the main manuscript, restricted to the Eyes and Mouth AOIs, reflected most of infants' gaze behavior in this group ($PTLT_{Eyestotal+Mouthtotal} = 72.7\%$).

For the 12-month-old bilinguals in the Lip-protrusion condition, results (AIC: 7058; BIC: 7090) revealed that infants looked significantly longer at the Eyes ($PTLT_{Eyestotal} = 14\%, SD = 2.8\%; Estimate = .61, SE = .17, t = 3.4, p < .001$) or at the Mouth AOIs ($PTLT_{Mouthtotal} = 70\%, SD = 7.6\%; Estimate = 6.3, SE = .22, t = 35.2, p < .001$) than at the Other AOI ($PTLT_{Other} = 8\%, SD = 5.5\%$). They however looked a similar amount of time at the Rest of the Face AOI ($PTLT_{ROFtotal} = 8\%, SD = 2\%; t < 1$) than at the Other AOI. In other words, these results show that the analyses presented in the main manuscript, restricted to the Eyes and Mouth AOIs, reflected most infants' gaze behavior in this group ($PTLT_{Eyestotal+Mouthtotal} = 84\%$).

12-month-old monolinguals

For the 12-month-old monolinguals in the Eyebrow-raise condition, results (AIC: 7546; BIC: 7578) revealed that infants looked significantly longer at the Eyes ($PTLT_{Eyestotal} = 18\%, SD = 4\%; Estimate = .98, SE = .22, t = 4.7, p < .001$) or at the Mouth AOIs ($PTLT_{Mouthtotal} = 57\%, SD = 9\%; Estimate = 2.8, SE = .22, t = 12.7, p < .001$) than at the Other AOI ($PTLT_{Other} = 13\%, SD = 7\%$). They however looked less time at the Rest of the Face AOI ($PTLT_{ROFtotal} = 12\%, SD = 3\%; Estimate = -.35, SE = .17, t = -2.1, p < .05$) than at the Other AOI. In other words, these results show that the analyses presented in the main manuscript, restricted to the Eyes and Mouth AOIs, reflected most infants' gaze behavior in this group ($PTLT_{Eyestotal+Mouthtotal} = 75\%$).

For the 12-month-old monolinguals in the Lip-protrusion condition, results (AIC: 7327; BIC: 7359) revealed that infants looked significantly longer at the Eyes ($PTLT_{Eyestotal} = 18\%, SD = 5\%; Estimate = .97, SE = .19, t = 4.9, p < .001$) or at the Mouth AOIs ($PTLT_{Mouthtotal} = 63\%, SD = 8\%; Estimate = 5.5, SE = .19, t = 28.4, p < .001$) than at the Other AOI ($PTLT_{Other} = 9\%, SD = 4\%$). They however looked a similar amount of time at the Rest of the Face AOI ($PTLT_{ROFtotal} = 10\%, SD = 3\%; t < 1$) than at the Other AOI. In other words, these results show that the analyses presented in the main manuscript, restricted to the Eyes and Mouth AOIs, reflected most infants' gaze behavior in this group ($PTLT_{Eyestotal+Mouthtotal} = 81\%$).

15-month-old bilinguals

For the 15-month-old bilinguals in the Eyebrow-raise condition, results (AIC: 7223; BIC: 7255) revealed that infants looked a similar amount of time at the Eyes ($PTLT_{Eyestotal} = 15\%, SD = 5\%; t < 1$) than at the Other AOI ($PTLT_{Other} = 15\%, SD = 7\%$). However, they clearly looked longer at the Mouth AOI ($PTLT_{Mouthtotal} = 65\%, SD = 10\%; Estimate = 5.1, SE = .19, t = 26.9, p < .001$) than at the Other AOI. They looked less time at the Rest of the Face AOI ($PTLT_{ROFtotal} = 5.6\%, SD = 3\%; Estimate = -.92, SE = .19, t = -4.9, p < .001$) than at the Other AOI. In other words, these results show that the analyses presented in the main manuscript, restricted to the Eyes and Mouth AOIs, reflected most infants' gaze behavior in this group ($PTLT_{Eyestotal+Mouthtotal} = 80\%$).

For the 15-month-old bilinguals in the Lip-protrusion condition, results (AIC: 7559; BIC: 7591) revealed that infants looked a similar amount of time at the Eyes ($PTLT_{Eyestotal} = 15\%, SD = 4\%; Estimate = .27, SE = .20, t = 1.3, p > .05$) than at the Other AOI ($PTLT_{Other} = 13\%, SD = 8\%$). However, they clearly looked longer at the Mouth AOI ($PTLT_{Mouthtotal} = 63\%, SD = 9\%; Estimate = 5.1, SE = .20, t = 24.2, p < .001$) than at the Other AOI. They looked a comparable amount of time at the Rest of the Face AOI ($PTLT_{ROFtotal} = 9\%, SD = 4\%; Estimate = -.37, SE = .20, t = -1.8, p > .05$) than at the Other AOI. In other words, these results show that the analyses presented in the main manuscript, restricted to the Eyes and Mouth AOIs, reflected most infants' gaze behavior in this group ($PTLT_{Eyestotal+Mouthtotal} = 78\%$).

15-month-old monolinguals

For the 15-month-old monolinguals in the Eyebrow-raise condition, results (AIC: 8381; BIC: 8413) revealed that infants looked longer at the Eyes ($PTLT_{Eyestotal} = 24\%, SD = 5\%; Estimate = 1.1, SE = .21, t = 5, p < .001$) and at the Mouth AOIs ($PTLT_{Mouthtotal} = 53\%, SD = 9\%; Estimate = 3.9, SE = .21, t = 19.1, p < .001$) than at the Other AOI ($PTLT_{Other} = 14\%, SD = 8\%$). They looked less time at the Rest of the Face AOI ($PTLT_{ROFtotal} = 9\%, SD = 4\%; Estimate = -.5, SE = .21, t = -2.5, p < .05$) than at the Other AOI. In other words, these results show that the analyses presented in the main manuscript, restricted to the Eyes and Mouth AOIs, reflected most infants' gaze behavior in this group ($PTLT_{Eyestotal+Mouthtotal} = 77\%$).

For the 15-month-old monolinguals in the Lip-protrusion condition, results (AIC: 7381; BIC: 7412) revealed that infants looked a similar amount of time at the Eyes ($PTLT_{Eyestotal} = 11\%, SD = 5\%; Estimate = -.24, SE = .20, t = -1.2, p > .05$) than at the Other AOI ($PTLT_{Other} = 14\%, SD = 9\%$). However, they clearly looked longer at the Mouth AOI ($PTLT_{Mouthtotal} = 66\%, SD = 8\%; Estimate = 5.2, SE = .20, t = 26.7, p < .001$) than at the Other AOI. They looked less time at the Rest of the Face AOI ($PTLT_{ROFtotal} = 9\%, SD = 4\%; Estimate = -.51, SE = .20, t = -2.6, p < .05$) than at the Other AOI. In other words, these results show that the analyses presented in the main manuscript, restricted to the Eyes and Mouth AOIs, reflected most infants' gaze behavior in this group ($PTLT_{Eyestotal+Mouthtotal} = 77\%$).

18-month-old bilinguals

For the 18-month-old monolinguals in the Eyebrow-raise condition, results (AIC: 8248; BIC: 8280) revealed that infants looked longer at the Eyes ($PTLT_{Eyestotal} = 29\%, SD = 9\%; Estimate = 1.6, SE = .19, t = 8.3, p < .001$) and at the Mouth AOIs ($PTLT_{Mouthtotal} = 48\%, SD = 14\%; Estimate = 3.5, SE = .19, t = 18.5, p < .001$) than at the Other AOI ($PTLT_{Other} = 11\%, SD = 7\%$). They looked a similar amount of time at the Rest of the Face AOI ($PTLT_{ROFtotal} = 12\%, SD = 4\%; t < 1$) than

at the Other AOI. In other words, these results show that the analyses presented in the main manuscript, restricted to the Eyes and Mouth AOIs, reflected most infants' gaze behavior in this group ($PTLT_{Eyestotal+Mouthtotal} = 77\%$).

REFERENCES

Gilet, E., Diard, J., and Bessière, P. (2011). Bayesian action-perception computational model: Interaction of production and recognition of cursive letters. *PLoS ONE* 6, e20387
